# Exposure to chemical cocktails before or after conception – The effect of timing on ovarian development^☆^

**DOI:** 10.1016/j.mce.2013.06.016

**Published:** 2013-08-25

**Authors:** Michelle Bellingham, Maria R. Amezaga, Beatrice Mandon-Pepin, Christopher J.B. Speers, Carol E. Kyle, Neil P. Evans, Richard M. Sharpe, Corinne Cotinot, Stewart M. Rhind, Paul A. Fowler

**Affiliations:** a Division of Applied Medicine, Institute of Medical Sciences, University of Aberdeen, Foresterhill, Aberdeen AB25 2ZD, UK; b Institute of Biodiversity, Animal Health and Comparative Medicine, College of Medical, Veterinary and Life Sciences, University of Glasgow, Glasgow G61 1QH, UK; c INRA, UMR 1198, Biologie du Développement et Reproduction F-78350, Jouy-en-Josas, France; d The James Hutton Institute, Craigiebuckler, Aberdeen AB15 8QH, UK; e MRC Centre for Reproductive Health, Queen’s Medical Research Institute, University of Edinburgh, 47 Little France Crescent, Edinburgh EH16 4TJ, UK

**Keywords:** Anti-ACTB, anti-β actin, DEHP, diethylhexylphthalate, ECs, environmental chemicals, EDCs, endocrine disrupting chemicals, FSH, follicle stimulating hormone, LH, luteinising hormone, WB, Western blot, Ovary, Development, In utero exposure, Environmental chemicals, Mixtures, EDCs

## Abstract

Exposure of female fetuses to environmental chemicals (ECs) during pregnancy results in a disturbed ovarian adult phenotype. We investigated the influence of pre- and/or post-conception exposure to low-level mixtures of ECs on the structure and function of the fetal ovine ovary. We examined ovarian morphology, expression of oocyte and granulosa cell-specific genes and proteome. Female fetuses were collected at day 110 of gestation, from dams exposed continuously until, and after mating, by grazing in pastures treated with sewage sludge as a fertiliser (TT) or in control fields treated with inorganic fertiliser (CC). In addition, in a cross-over design, fetal ovaries were collected from dams maintained on sludge pastures up to the time of mating but then transferred to control pastures (TC) and, reciprocally, those transferred from control to treated pastures at mating (CT). On examination, the proportion of type 1a follicles (activating primordial follicles) was significantly lower in animals from the CT groups compared with CC and TT groups (*P* < 0.05). Of the 23 ovarian gene transcripts studied, 14 were altered in the ovaries of exposed fetuses (CT, TC, and TT) relative to controls, with the largest number of changes observed in cross-exposure pattern groups (CT or TC). Continuous EC exposure (TT) produced fewer transcript alterations and only two genes (*INHBA* and *GSN*) presented differential profiles between CC and TT. Fetal ovarian proteome analysis (2-DE gels) showed, across all exposure groups, 86 differentially expressed protein spots compared to controls. Animals in the CT group exhibited the highest number (53) while TC and TT presented the same number of affected protein spots (42). Fetal ovarian proteins with altered expression included MVP (major vault protein) and several members of the heat-shock family (HSPA4L, HSP90AA1 and HSF1). The present findings indicate that continuous maternal EC exposure before and during gestation, are less deleterious for fetal ovarian development than a change in maternal EC exposure between pre and post-conception. The pathways by which the ovary responds to this chemical stress were common in TT, CT, TC exposed foetuses. In addition to the period of pregnancy, the pre-conception period appears also as crucial for conditioning long-term effects of EC exposure on ovarian development and primordial follicle reserve and hence future fertility.

## Highlights

• In-utero exposure to environmental chemicals disturbs ovary development.
• We investigated differential effects of exposure before or after conception.
• The fetal ovary is most affected by exposure after conception.
• Unexpectedly, response to continuous exposure was less severe than previously.
• Alterations in profiles of in utero exposure to chemicals may be most damaging.

## 1 Introduction

Environmental chemicals (ECs), including endocrine disrupting compounds (EDCs), adversely affect multiple physiological systems in a wide range of animal species (Colborn et al., 1993; Fowler et al., 2012; Magnusson, 2012; Rhind, 2005). Effects on reproductive function of controlled exposures, to large amounts of single chemicals, have been studied using laboratory rodents or cell cultures (Craig et al., 2011; Gray et al., 2000; Meerts et al., 2001), while in other studies, abnormalities have been retrospectively linked to environmental exposure to abnormally high levels of individual pollutants (Guillette et al., 1994; Wolfe et al., 1995). Using sheep as an experimental model and sewage sludge applications to pasture as a means of exposing them to environmental levels of a mixture of ECs, the relationships between “everyday”, background EC exposure, associated tissue chemical burdens and physiological status have begun to be addressed. These studies have shown that small increases in tissue concentrations of selected ECs (Rhind, 2002, Rhind et al., 2005), were associated with perturbations of hypothalamo-pituitary (Bellingham et al., 2009, 2010; Rhind et al., 2010a) testicular (Bellingham et al., 2012; Paul et al., 2005) and ovarian physiology (Fowler et al., 2008) in the offspring of sludge-exposed mothers. Whilst these animals had been continually exposed to sludge treated pastures, and thus theoretically ECs, grazing at different sites and changes in environmental conditions probably means that such EC exposure is not at a constant level over an animal’s lifetime. It has been previously shown that among ewes reared in fields showing accumulation of phthalates, the percentage of animals with presence of DEHP increases with age. This is due in part to a longer exposure but it likely also due to the mobilization of body reserves which occurs during pregnancy (Herreros et al., 2010). Therefore, fetuses may only be exposed to biologically significant concentrations of ECs during specific periods of development and the physiological response associated with exposure may be influenced by the timing and extent of placental and fetal hepatic biotransformation of ECs (e.g. O’Shaughnessy et al., 2011). The sensitivity of a fetus to ECs may also differ depending on the time/stage of development.

In addition, the level and composition of the chemical mixture to which animals are exposed may differ according to its origin, i.e. through environmental/dietary exposure or as a result of maternal tissue mobilisation during gestation (Bigsby et al., 1997). In an extension of the ovine studies indicated above, we examined whether such differences in the profile, timing and/or rate of EC exposure might influence the physiological responses induced in the exposed animals, in particular, how did the timing of maternal, and thus fetal exposure, affect their respective tissue EC concentrations? Tissue EC concentrations were assessed in 3 different groups of ewes and their offspring where the mothers were exposed to sludge-treated pastures during different life stages: (1) throughout life (fetal exposure to ambient and maternally stored ECs), (2) before mating, but not thereafter (fetal EC exposure primarily attributable to release from mobilised tissue), (3) during the first 110 days of gestation (principally ambient exposure), a period of rapid tissue differentiation and development in the fetus which were then compared to unexposed (control) animals. Despite very few differences in EC profiles in either maternal or fetal sludge-exposed tissues, many previous studies using this model (Rhind et al., 2011, 2009, 2010b) have shown that the absence of significant differences in measured tissue burden at slaughter is not necessarily indicative (or predictive) of the previous pattern of exposure or of the occurrence of physiological effects.

The present paper reports the results of extensive investigations into effects of maternal exposure to sludge-treated pastures on fetal ovarian physiology, in three exposure groups and a control group maintained on sludge free pasture throughout life outlined above. Effects on fetal ovarian physiology were investigated by means of changes in fetal ovarian histomorphology, endocrinology, the proteome and the transcription of essential genes for fetal ovarian development.

## 2 Material and methods

### 2.1 Ethics statement

All animals used in this study were treated humanely with due consideration to the alleviation of pain, suffering, distress or lasting harm according to the James Hutton Institute’s (formerly the Macaulay Land Use Research Institute) Local Ethical Committee and fully licensed by the United Kingdom’s Animals (Scientific Procedures) Act 1986 under Project License authority (60/3356). Project license approval automatically includes a prior ethical committee evaluation and approval process, is legally binding, and a legal necessity. All in vivo components of the study were conducted at the James Hutton Institute under this legal framework operating at the highest ethical standards. Therefore, separate ethics approvals from the individual research institutions receiving ex-vivo tissue samples (University of Glasgow, University of Aberdeen, INRA and MRC Centre for Reproductive Biology) are superseded.

### 2.2 Experimental animals, management and monitoring

**Fig. 1:**
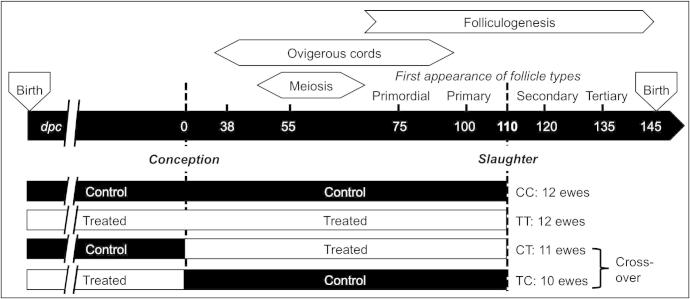
Diagrammatic summary of study design. Adults ewes were maintained on either control (inorganic fertiliser) or exposed (sewage sludge fertiliser) pastures until mating The CC and TT ewes continued on the same pastures, but the cross-over ewes, TC, CT, were moved to the opposite exposure pastures. The CC ewes were never exposed to sewage sludge while the TT ewes were always exposed to sewage sludge. The TC ewes were exposed to sewage sludge only before mating while the CT ewes were only exposed to sewage sludge after mating.

The experimental design has been described previously (Hombach-Klonisch et al., 2013; Rhind et al., 2010b) and is summarised in Fig. 1. Briefly, groups of Texel ewes from a single flock were maintained at conventional stocking rates at the James Hutton Institute’s research station at Hartwood in Scotland. Pastures were fertilised twice annually (early spring and late summer) with either thermally dried digested sewage sludge (2.25 metric tons of dry matter/ha; Treated; T) or inorganic fertiliser containing equivalent amounts of nitrogen (225 kg /ha/year; Control; C). Ewes of all experimental groups were mated to Texel rams at a synchronised oestrus during the normal breeding season (November). Ewes were randomly assigned to one of 4 experimental groups (*n* = 12/group). Two groups were exposed to either the sludge-treated (TT) or control (CC) pastures, throughout their breeding lives up until the time of slaughter at 110 days gestation. Two additional groups of ewes (*n* = 12/group), one of which had been maintained on sludge-treated and one on control pastures, were then swapped onto, and maintained on, the opposite pasture type up until slaughter at 110 days gestation giving rise to CT and TC groups (Fig. 1). Ewes from the CT and TC groups were mated approximately 2 weeks after the CC and TT ewes for enforced husbandry reasons (Additional Information found in Appendix A). Day 110 of gestation was selected for tissue collections since the sheep fetal ovary contains the main classes of follicles by this stage of development: primordial, primary, preantral and few small antral (Fowler et al., 2008; McNatty et al., 1995; Sawyer et al., 2002) at this time.

### 2.3 Tissue collection

At slaughter, maternal and fetal liver (from one fetus/ewe) was collected for determination of EDC content, as reported previously (Rhind et al., 2010b). Fetal ovaries were either snap-frozen in liquid nitrogen for mRNA extraction or fixed for 5 h in Bouins solution before storage in 70% ethanol prior to processing to wax for histological analysis. Fetal and maternal blood samples were also collected and the plasma stored at −20 °C for hormone measurements.

### 2.4 Hormone assay

Plasma estradiol concentrations were estimated in duplicate, diethyl ether extracts of 200 μl of plasma (3 assays) using a modification (Evans et al., 1994) of the Serono Estradiol MAIA assay (Serono-Baker Diagnostics, Inc., Allentown, PA). Mean intra- and inter-assay coefficients of variation (CV) were 8.5% and 6.15% respectively and assay sensitivity averaged 0.27 pg/ml. Plasma concentrations of follicle-stimulating hormone (FSH) and luteinizing hormone (LH) were measured, in duplicate samples (0.1–0.2 ml), by radioimmunoassay that has been described and validated previously for sheep (McNeilly et al., 1986); the assay standards used were NIDDK-FSH-RP2 and NIH-LH-S12, and assay sensitivities were 0.1 and 0.2 ng/mL for FSH and LH, respectively. Mean intra-assay CV for LH was 7.5%. Plasma testosterone concentrations were measured, in duplicate, after extraction of samples (0.2 ml) with diethyl ether using a modification of a previously-described protocol (Sheffield and O’Shaughnessy, 1989). Mean intra- and inter-assay CV were 9.4% and 9.6% respectively over three assays and assay sensitivity averaged 0.015 ng/ml. Plasma levels of inhibin A (INHA) were measured using a two-site, enzyme-linked immunoassay that uses a capture antibody directed against amino acid sequence 82–114 of the human and ovine β-A subunit and a C-specific biotinylated monoclonal antibody raised against a synthetic peptide that corresponds to amino acid sequence 1–32 of the human α-C subunit, as the detection antibody (Knight et al., 1998). Plasma levels of progesterone were measured with the automated ADVIA Centaur® XP competitive immunoassay system, which uses direct chemiluminescent detection (Siemens Healthcare Diagnostics, Camberley, UK). Mean intra- and inter-assay CV were 1.9% and 3.2% respectively and assay sensitivity 0.67 nmol/l. Measurements were performed on serial dilutions of several randomly selected samples to ensure that all values within the range of dilutions were within the limits of detection; dilutions produced proportional and linear results.

### 2.5 Ovarian histomorphology and immunohistochemistry

**Fig. 2:**
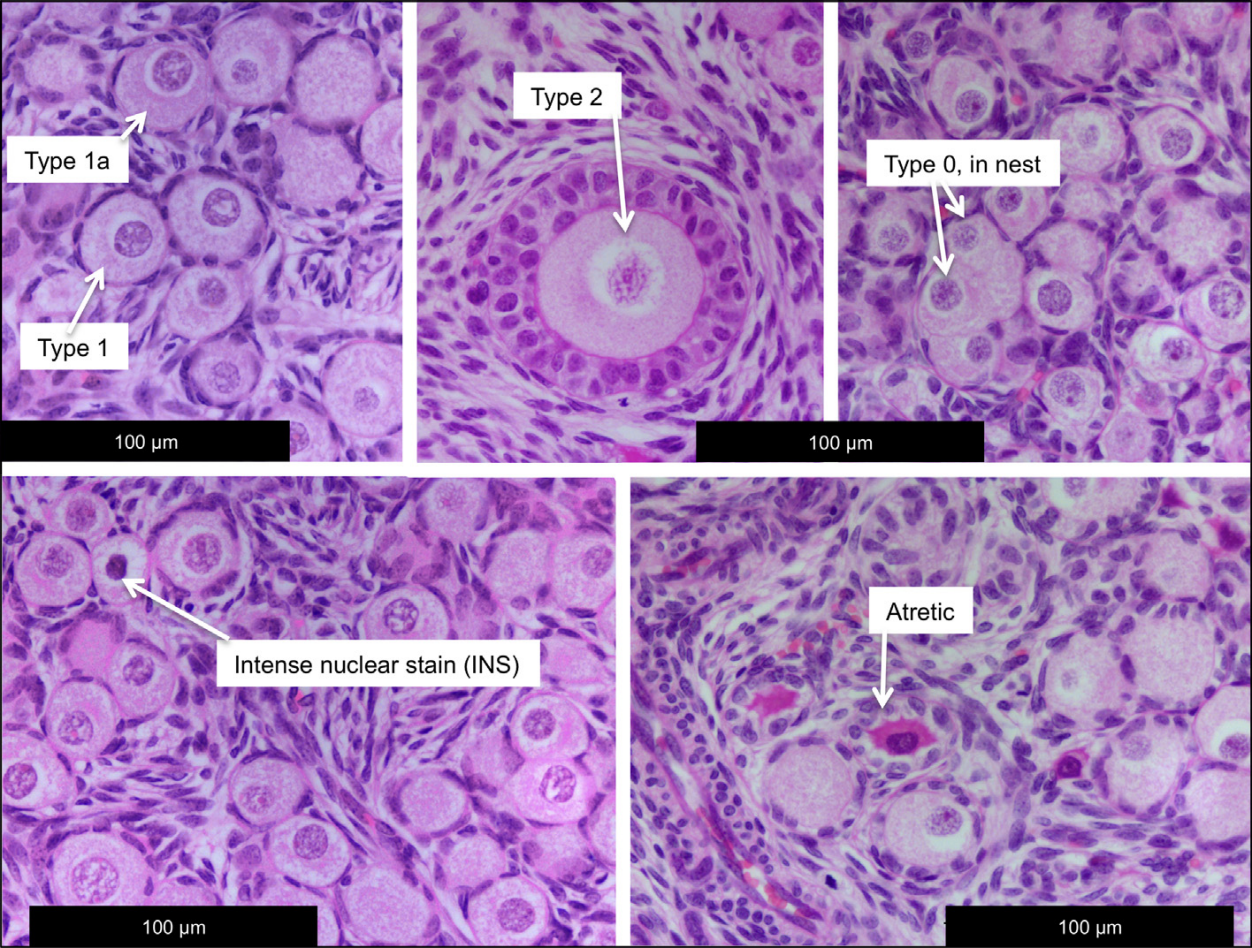
Representative oocyte and follicle classification used to quantify the morphological effects of sewage sludge exposures on the fetal sheep ovary. The figure shows typical examples of oogonial nests, types 0, 1, 1a and 2 follicles (healthy) and also intense nuclear staining and follicular atresia.

Using a modified protocol from our previous study (Fowler et al., 2008), ovarian sections were analysed for oocyte numbers and follicle size classes using an established follicle classification system (Lundy et al., 1999). Briefly, 5 μm sagittal sections of each fetal ovary were cut and stained with H&E. For all ovaries, slides were prepared containing two randomly selected, non-consecutive sections. Four randomly selected ovarian H&E sections, >50 μm apart, were examined by a single assessor using 6 fields of view per section at *x*10 objective magnification. Only oocytes with the nucleus clearly visible were included in quantification of oocyte and follicle densities. The incidences of atresia and heavily stained, condensed, oocyte nuclei were also quantified and are shown in Table 1 and representative images are shown in Fig. 2. For immunohistochemistry, 5 μm sections were mounted onto Superfrost slides and immuno-stained using a BOND-MAX™ automated immunostainer (Leica Microsystems Newcastle Ltd., Newcastle upon Tyne, UK). Immunohistochemistry was performed as previously described (Fowler et al., 2009) using the Bond Polymer Refine detection kit (Leica Biosystems, Newcastle, UK) following manufactures instructions. Where applicable, the Bond DAB Enhancer (Leica Microsytems Newcastle, UK) was also included to maximise the contrast between chromogen-specific staining and the counterstain. Microwave (2 × 5 min, high power) citrate (pH 6.0) antigen retrieval was used for all antibodies (except for HSPA4L, which did not require antigen retrieval). Antibodies used were: (i) HSP60 1:25, mouse (ab1819, Abcam); (ii) HSP70 1:800, mouse (ab47455, Abcam); (iii) HSP90 1:500, mouse (H1775, Sigma–Aldrich); (iv) HSPA4L 2.5 μg/ml, goat (LS-B2621, Life Span Biosciences Inc.); (v) HSF1 1:75, rabbit (ab47484, Abcam); (vi) MVP 1:200, mouse (ab14562, Abcam); (vii) DNASE1 1:1000, rabbit (18-732-292145 GenWay); (viii) ANXA1 1:125, goat (LS-B3028/26040, Life Span Biosciences Inc.); (ix) CDKN1B 1:100, rabbit (GTX27961, GeneTex Inc.); (x) IDH1 1:100, rabbit (ab36329, Abcam); (xi) GSTM3 1:100, rabbit (15214-1-AP, ProteinTech) which were incubated for 30 min at room temperature. Negative control sections incubated with non-immune serum were included and showed no staining.

### 2.6 Fetal ovarian protein and RNA extraction

For proteomic analysis, proteins were extracted from fetal ovaries using a Qiagen AllPrep DNA/RNA/Protein mini kit (Qiagen Ltd., Crawley, UK; cat. No. 80004) following tissue homogenization using a Qiagen TissueLyser (Qiagen Ltd., Crawley, UK; cat No. 85300). Manufacturer’s instructions were followed with two modifications: (i) the addition of protease inhibitors (Protease Inhibitor Cocktail, Sigma–Aldrich Company Ltd., Gillingham, UK; cat. No. P8340) to the lysis buffer (RLT) to maximise protein integrity and yield and (ii) the re-suspension of protein pellets in Modified Reswell Solution (MRS; 7M urea, 2M thiourea, 4% (w/v) CHAPS, 0.3% (w/v) DTT) to facilitate protein solubilisation. Total RNA was extracted from fetal ovaries using TRIzol LS reagent (Invitrogen, Cergy Pontoise, France). RNA yield and quality were determined spectrophotometrically (Nanodrop ND1000 LABTECH). To avoid contaminating DNA, 30 μg of each sample were treated with 30U of RNase-Free DNase Set (Qiagen, Les Ulis, France) and then purified with RNeasy MiniElute Cleanup kit (Qiagen, Les Ulis, France).

### 2.7 Quantitative RT-PCR (qPCR) analysis

Extracted RNA, was reverse transcribed to cDNA as described previously (Mandon-Pepin et al., 2003). Briefly, 2 μg DNase-treated RNA was reverse transcribed in a volume of 20 μL Superscript II RNase H-reverse transcriptase (Invitrogen, Cergy Pontoise, France). qPCR analysis was performed using the ABI Prism 7700 HT apparatus (Applied Biosystems) and ABsolute blue QPCR SYBR Green ROX mix (Abgene, Les Ulis, France), using 5 ng of cDNA as a template. The primers used for real-time PCR are shown in Appendix B. Samples were each run in triplicate and the median value was used for analysis. PCR efficiencies were calculated from gene-specific standard curves. Data were normalized against expression of the house-keeping gene HPRT1 and expressed as percentages of maximum expression and the SEM was calculated from both independent qPCR reactions.

### 2.8 Proteomics

Proteins in protein pools, to which individual ovaries contributed equally, were separated by 2-DE in quadruplicate using commercial 7 cm gels, as previously described (Fowler et al., 2008); gels were stained with Colloidal Coomassie Brilliant Blue, and scanned (Ettan DIGE Imager, GE Healthcare). Gel images were analysed and protein spots detected and quantified using Progenesis SameSpots software, V 6.01 (Nonlinear Dynamics, Newcastle, UK). (Silva et al., 2010). The software was used to combine the gel quadruplicates and calculate fold-changes and significance (by ANOVA of log-normalised values). Differentially expressed protein spots were selected for spot excision once these ANOVA values had been independently verified by the authors (see Statistical Analysis below). Proteins in the gel pieces were digested with trypsin (sequencing grade, modified; Promega UK, Southampton, UK) using an Investigator ProGest robotic workstation (Genomic Solutions Ltd., Huntingdon, UK). Peptide solutions were analyzed using an HCTultra PTM Discovery System (Bruker Daltonics Ltd., Coventry, UK) coupled to an UltiMate 3000 LC System (Dionex (UK) Ltd., Camberley, Surrey, UK). Peptides were separated on a Monolithic Capillary Column (200 μm i.d. × 5 cm; Dionex part No. 161409). Peptide fragment mass spectra were acquired in data-dependent AutoMS(2) mode with a scan range of 300–1500 m/z, 3 averages, and up to 3 precursor ions selected from the MS scan 100–2200 m/z). Precursors were actively excluded within a 1.0 min window, and all singly charged ions were excluded. Peptide peaks were detected and deconvoluted automatically using Data Analysis software (Bruker). Mass lists in the form of Mascot Generic Files were created automatically and used as the input for Mascot MS/MS Ions searches of the NCBInr database using the Matrix Science web server (www.matrixscience.com). The default search parameters used were: Enzyme = Trypsin, Max. Missed cleavages = 1; Fixed modifications = Carbamidomethyl (C); Variable modifications = Oxidation (M); Peptide tolerance ± 1.5 Da; MS/MS tolerance ± 0.5 Da; Peptide charge = 2+ and 3+; Instrument = ESI-TRAP. Statistically significant MOWSE scores and good sequence coverage were considered to be positive identifications.

### 2.9 1-D Gel electrophoresis and Western blot (WB)

Individual ovary protein extracts were electrophoresed (30 μg protein/lane) along with Odyssey Two-Colour Protein Molecular Weight Markers (Li-COR Biosciences UK Ltd., Cambridge, UK) on 26-lane 1-DE 4–12% Bis-Tris gels (Invitrogen Ltd., Paisley, UK) under reducing conditions (MOPS buffer, Invitrogen) and transferred to Immobilon™-FL membranes (Millipore (UK) Ltd., Watford, UK) as described previously (Fowler et al., 2008). Membranes were blocked (1 h, room temperature) with Odyssey Blocking Buffer, (927–4000: LICOR + PBS) before incubation with the following mouse monoclonal primary antibodies at 4 °C overnight: (i) Heat shock protein 60 (HSP60), 1:1,000 (ab1819, Abcam Cambridgeshire, UK); (ii) HSP70, 1:1,000 (ab47455, Abcam); (iii) HSP90, 1:1,000 (H1775, Sigma–Aldrich,Dorset, UK); (iv) Major Vault Protein (MVP), 1 μg/ml (ab14562, Abcam); Gelsolin (GSN) 3 μg/ml (ab55070, Abcam); (v) HSP90, 1:1,000 (Sigma–Aldrich, UK) (vi) and goat HSPA4L 1 μg/ml, (LS-B2621, Life Span Biosciences Inc, Seattle,USA); (vii) PTEN 2 μg/ml (ab24367, Abcam). Membranes were also incubated with an anti-ACTB (anti ß-actin) as a loading control (1:5,000, mouse, ab8226, Abcam or 1:10,000, rabbit A5060, Sigma–Aldrich). IRDye® infrared secondary antibodies (Li-COR) were incubated after primary antibody incubation then protein bands were visualized using the Li-COR Odyssey® Infrared Imaging System. Band images were analyzed using TotalLab TL120 software (v2008.1; Nonlinear Dynamics Ltd., Newcastle-upon-Tyne, UK) to determine the molecular weights and band volumes.

### 2.10 Statistical analysis and bioinformatics

Analyses were performed using JMP (7.02, Thomson Learning, London, UK). Normality of data distribution was tested with the Shapiro–Wilk test and, where distribution of data was skewed, they were log-transformed prior to analysis. Morphological and endocrine data and the 1-DE WB band volumes (normalized relative to ACTB expression separate for each lane) and normalized spot volumes (% of total spot volume for each gel separately) were compared in control and sewage sludge-exposed groups, using one-way ANOVA. Protein spots on the gels were assessed primarily by visual reproducibility and fold-change. Statistically significant differences (ANOVA) in log-transformed, normalized volumes were an important determinant of the spots selected for identification by LC/MS-MS. Unless stated otherwise, data are presented as mean ± s.e.m. Where fold-changes are presented a +ve value indicates an increase, and a −ve value a reduction, relative to controls. Proteins and transcripts exhibiting treatment-specific alterations in expression were analyzed using IPA version 9.0 (Ingenuity Systems, http://www.ingenuity.com), including canonical pathway analysis, functional network analysis and generation of graphical representation of networks (http://www.ingenuity.com/company/pdf/Citation_Guidelines_2005-09-13.pdf), as previously used by us to analyse human fetal ovarian gene array data.

## 3 Results

### 3.1 Maternal physiological measurements and endocrinology

There were no differences in the number or sex ratios of fetuses produced between the 4 groups. At slaughter, on day 110 of gestation, group differences in live weight were small and likely to be attributable to contemporary differences in gut fill. The mean condition scores were indicative of a normal nutritional state for the stage of gestation in all groups (Appendix C). At 110 days gestation when the ewes were slaughtered, the ovaries of the TC ewes were significantly heavier than the CC ewes (*P* < 0.05). The different EC exposure patterns also had subtle effects on maternal endocrinology at day 110 of gestation: TT ewes had significantly (*P* < 0.05) higher circulating progesterone than CC or CT, and significantly (*P* < 0.05) higher testosterone concentrations than CT ewes. The TC ewes had significantly (*P* < 0.05) lower LH than CC and TT ewes and significantly higher (*P* < 0.05) progesterone concentrations compared to control ewes. There were no significant effects of exposure on maternal estradiol, inhibin A or FSH concentrations (Appendix C).

### Fetal morphology, endocrinology

Fetal body and uterine weight did not differ significantly between the CC and EC exposed animals (Table 1). However there were some differences between groups in fetal endocrine variables, TT fetuses had higher circulating concentrations of inhibin A (*P* < 0.05) than the CC fetuses and higher FSH (*P* < 0.05) and lower estradiol (*P* < 0.05) than CT fetuses (Table 1). LH, testosterone and progesterone concentrations were not different between sludge-exposed groups and controls (Table 1).

### 3.2 Fetal ovarian characteristics

**Fig. 3:**
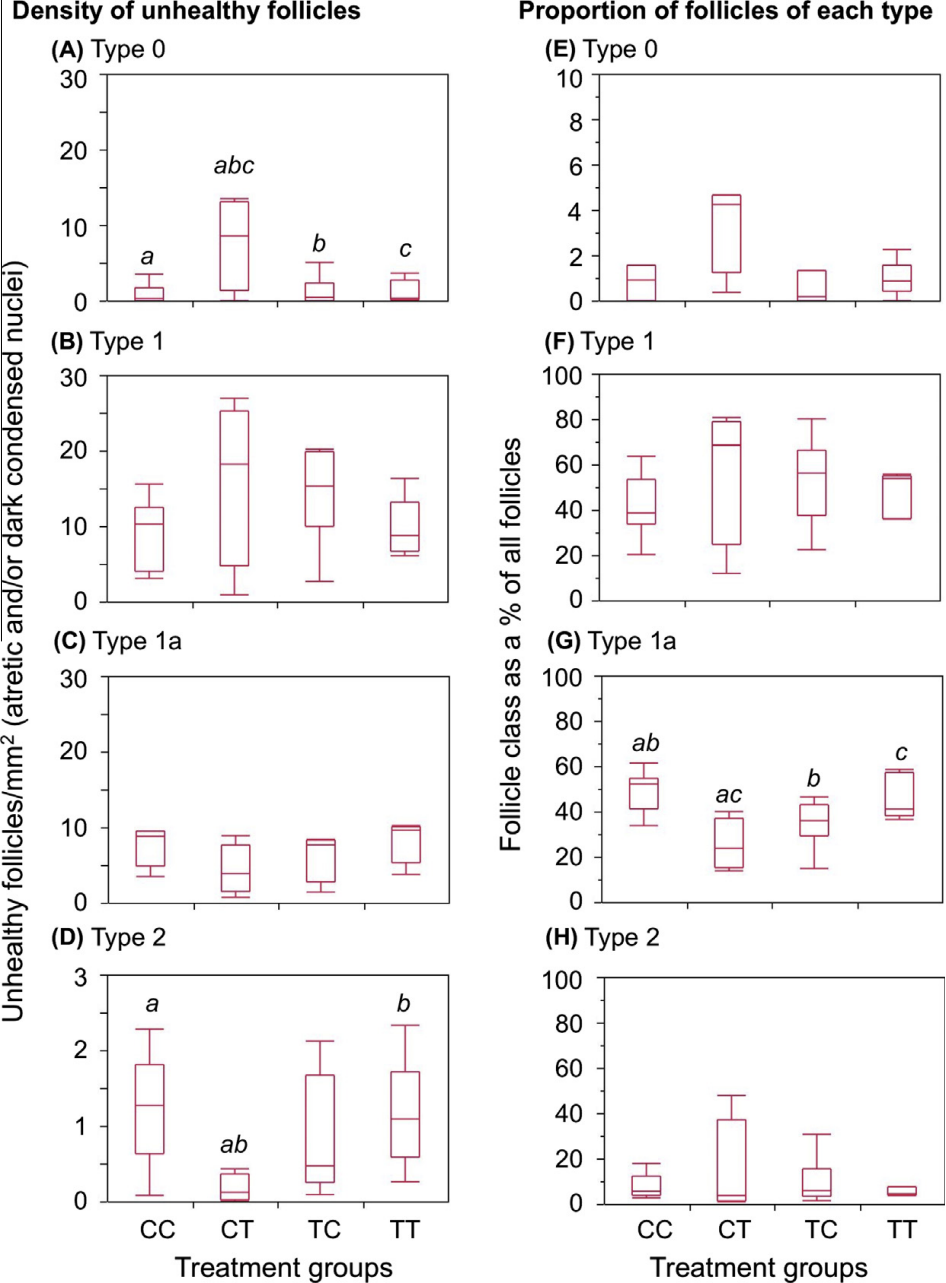
Exposure to sewage sludge from conception onwards alters fetal ovarian follicle characteristics. The densities of unhealthy follicles (A–D) classed as those with condensed, intensely stained nuclei and/or atresia are shown combined, while the relative proportions of all follicles of each class, relative to total follicle density (E–H) are shown in box and whisker plots. The horizontal line in the boxes show the median values, with the limits of the boxes showing the 25% and 75% quantiles and the whiskers showing the 10% and 90% quantiles. Common superscripts between groups, for each follicle type, denote statistically significant differences at *P* < 0.05.

All fetuses of sludge-exposed groups tended to have heavier ovaries than controls (CC), although this only reached statistical significance in the TT group (Table 1). In the subset of fetal ovaries that were examined histologically, the density of Type 0 oocytes (naked oocytes), and the densities of Type 1 (primordial) and Type 2 (primary) follicles (see Table 1, Fig. 2. (Lundy et al., 1999)) were similar in all groups (Fig. 3) However, the density of Type 1a (transitory) follicles in the CT group was less than half those in the CC and TT groups (*P* < 0.05). A similar, but non-significant, trend was observed in the TC fetal ovaries (Table 1). To elucidate the relative changes in follicle types, we compared differences in the proportions of each follicle type between control and exposure groups. Type 1a follicles formed a smaller proportion of total follicle density in the CT group than in controls (*P* < 0.05) and the TT group (*P* < 0.05) while in the TC group it was also lower than in controls (*P* < 0.05, Fig. 3G). The density of unhealthy Type 0 oocytes (defined as those with morphological characteristics of atresia, with or without highly condensed and intensely stained nuclei) was higher in the CT group compared with all other groups (*P* < 0.05, Fig. 3A). In conclusion, CT exposed ovaries presented the most phenotypic alterations in term of density, proportion and quality of Types 0 and 1a follicles.

### 3.3 Fetal ovarian proteome

**Fig. 4:**
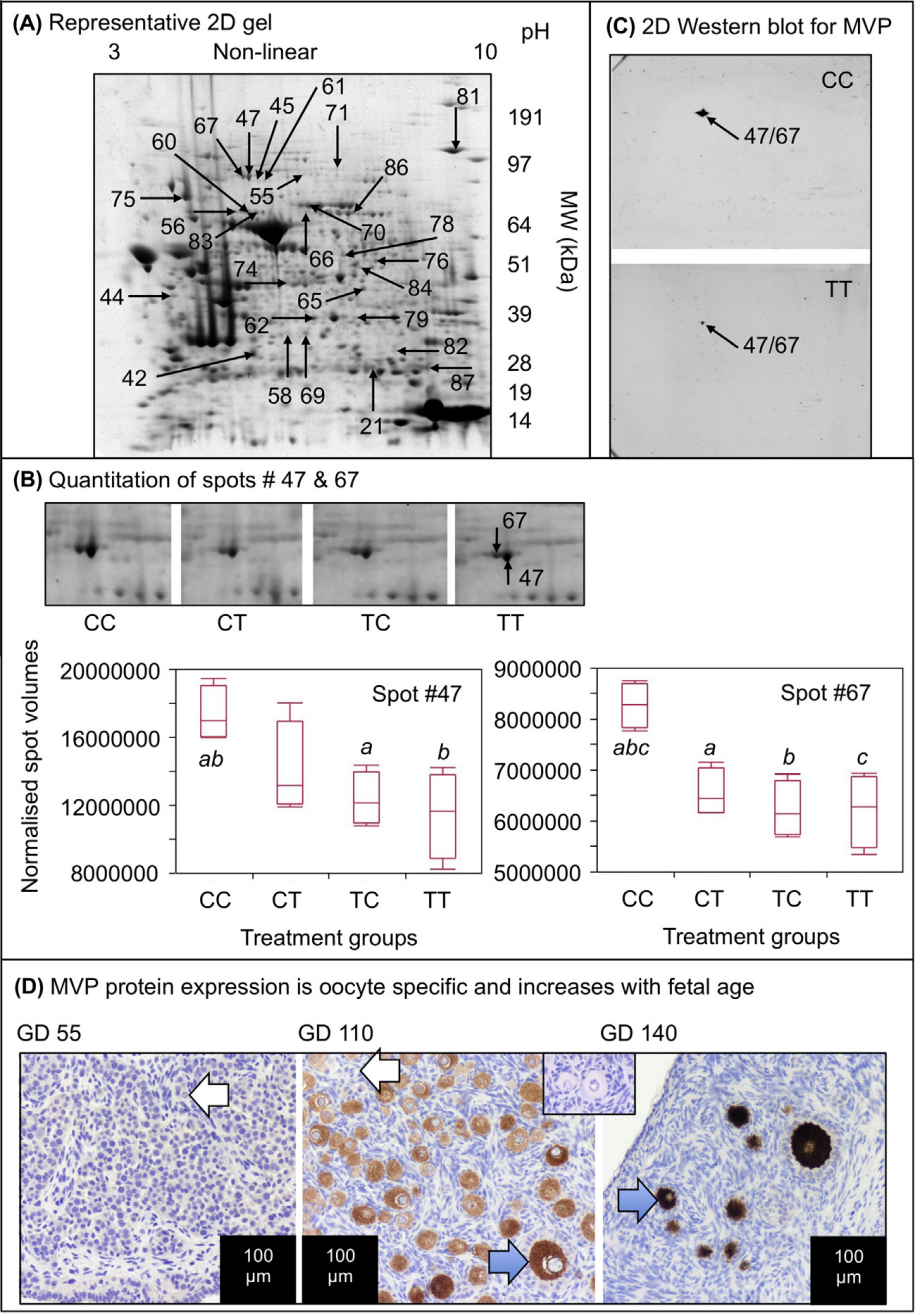
Representative proteomic data. (A) Representative 2-DE gel of CC group ovarian proteins is shown, with the spots identified in Table 3 denoted by arrows and numbers. (B) Quantitation of spots 47 and 67, both identified as MVP. Representative zoom-boxes for each treatment group are shown, as well as box and whisker plots of normalised spot volumes. The horizontal lines in the boxes show the median values, with the limits of the boxes showing the 25% and 75% quantiles and the whiskers showing the 10% and 90% quantiles. Common superscripts between groups, for each protein spot, denote statistically significant differences at *P* < 0.05. (C) Representative, not-quantitative 2-DE Western blots for MVP showing the localisation of a single spot at the locus of spot #47. D. Representative immunohistochemistry showing that MVP expression increases dramatically between day 55 and 140 of gestation in the fetal sheep ovary and is oocyte-specific. The inset box shows IgG-ve staining control.

**Fig. 5:**
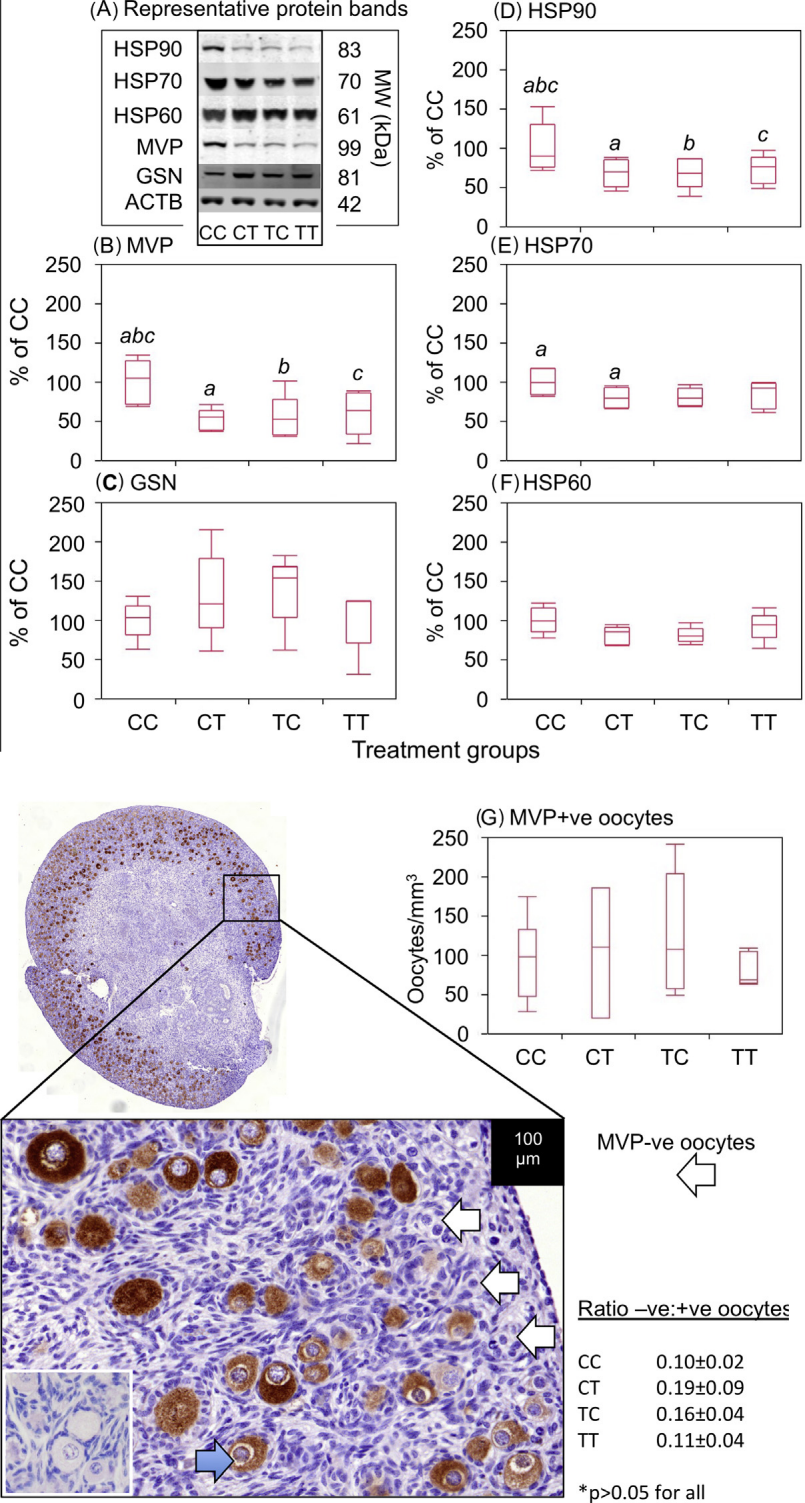
Sewage sludge exposure affects MVP, HSP90 and HSP70 proteins in fetal ovaries, quantified by Western blot. (A) Representative bands for each treatment group for each Western blot, including β-actin load control. These bands are all from the same 4 ovaries and all available ovaries were used for these Western blots. (B–F) Quantitation of 5 ovarian proteins shown as box and whisker plots. The band volume for each protein was normalised against β-actin for the same lane (i.e. same ovary) and then expressed relative to the mean normalised band volume of the CC treatment group. The horizontal line in the boxes show the median values, with the limits of the boxes showing the 25% and 75% quantiles and the whiskers showing the 10% and 90% quantiles. Common superscripts between groups, for each protein, denote statistically significant differences at *P* < 0.05. Where there are no superscripts p values are >0.05. (G) Quantification of MVP immunopositive and immunonegative oocytes shows that there were no significant exposure effects on the density of MVP immunopositive oocytes or the ratio between immunonegative and immunopositive oocytes, although the latter tended to be higher in the CT and TC groups.

Given the changes in follicle density and type between exposure groups, we were interested to determine which pathways are involved in these ovarian phenotypic modifications and to examine how exposure affected fetal ovarian protein expression. 509 fetal ovarian protein spots were considered suitable for inclusion in the statistical analysis of the 2-DE gel results, on the basis of their reproducibility across gels and, of these, 90 showed a statistically significant difference in normalised spot volumes between at least 2 of the experimental groups and 86 in relation to the control group (CC). The greatest number of differentially expressed spots, relative to the control group was in the CT group (53 spots *P* < 0.05) while the TC (42 spots; *P* < 0.05) and TT (42 spots; *P* < 0.05) exposure groups exhibited a similar number of differentially expressed spots (Table 2). 28 protein spots met the criteria for excision and identification by LC–MS/MS (Fig. 4A). The identities of the proteins successfully matched to the peptides recovered from these spots are shown in Table 3. Of the 20 proteins identified in spots differentially-expressed, 18 were altered in the CT (10 down-regulated) group while 11 were altered in both TC (8 down-regulated) and TT (7 down-regulated) groups compared to controls (*P* < 0.05). Three of the identified proteins were differentially expressed in all exposure groups, in comparison to controls: major vault protein (MVP), heat shock 70 kDa protein 4L (HSPA4L) and deoxyribonuclease-1 (DNASE1) (all down-regulated). MVP was further investigated because it showed statistically significant reductions in expression across the 3 exposure groups. This protein was identified in spots 47 and 67 in 2-DE gels; spot images and their corresponding spot volume analyses are shown in Fig. 4A and B. LC–MS/MS identification of MVP was confirmed by 2-DE Western blot (WB: Fig. 4C) with the immuno-detected protein overlapping the two spots. MVP expression was oocyte specific and increased from undetectable early in gestation to very heavy staining of oocytes at 140 days of gestation (Fig. 4D). MVP expression was validated by 1-DE WB (Fig. 5A and B, see Section 3.5). We went onto count MVP immunopositive and immunonegative oocytes in whole ovary scans and found no change in the densities of MVP-positive oocytes (Fig. 5G). Although there was a tendency for the CT and TC groups to have a higher proportion of MVP-negative oocytes, variability between fetuses meant that this was not statistically significant.

In the CT and TT groups heat shock protein 90-alpha (HSP90AA1) was down-regulated and heat shock factor protein 1 (HSF1) and alpha-fetoprotein (AFP) were up-regulated. Of the six proteins differentially expressed in both the CT and TC groups, lamin A/C (LMNA), aldose reductase (AKR1B1) and annexin A1 (ANXA1) were down-regulated and proteasome subunit alpha type-4 (PSMA4), glutathione S-transferase Mu 1 (GSTM1) and fibrinogen beta chain (FGB) were up-regulated. Five proteins were differentially expressed in the CT group exclusively: glutathione S-transferase Mu 3 (GSTM3) and dihydrolipoyl dehydrogenase (DLD) were down-regulated while heterogeneous nuclear ribonucleoprotein H3 (HNRNPH), vimentin (VIM) and aflatoxin B1 aldehyde reductase member 2 (AKR7A2) were up-regulated. In the TT group, exclusively, two proteins were differentially expressed: isocitrate dehydrogenase [NADP] (IDH1) (down-regulated) and septin 11 (SEPT11) and heterogeneous nuclear ribonucleoprotein H1 (HNRNPH1) (up-regulated) (Table 3).

The differentially-expressed, proteins fell into 5 major molecular/cellular functional categories (Table 5): (i) small molecule biochemistry (13 proteins), (ii) post-translational modification (3 proteins), (iii) protein folding (3 proteins), (iv) drug metabolism (5 proteins) and (v) lipid metabolism (8 proteins). These proteins fell into two functional networks (IPA analysis): (i) cancer, gastrointestinal disease, cellular movement (score 27) and (ii) cancer, genetic disorder, respiratory disease (score 26).

### 3.4 Gene expression profiling

**Fig. 6:**
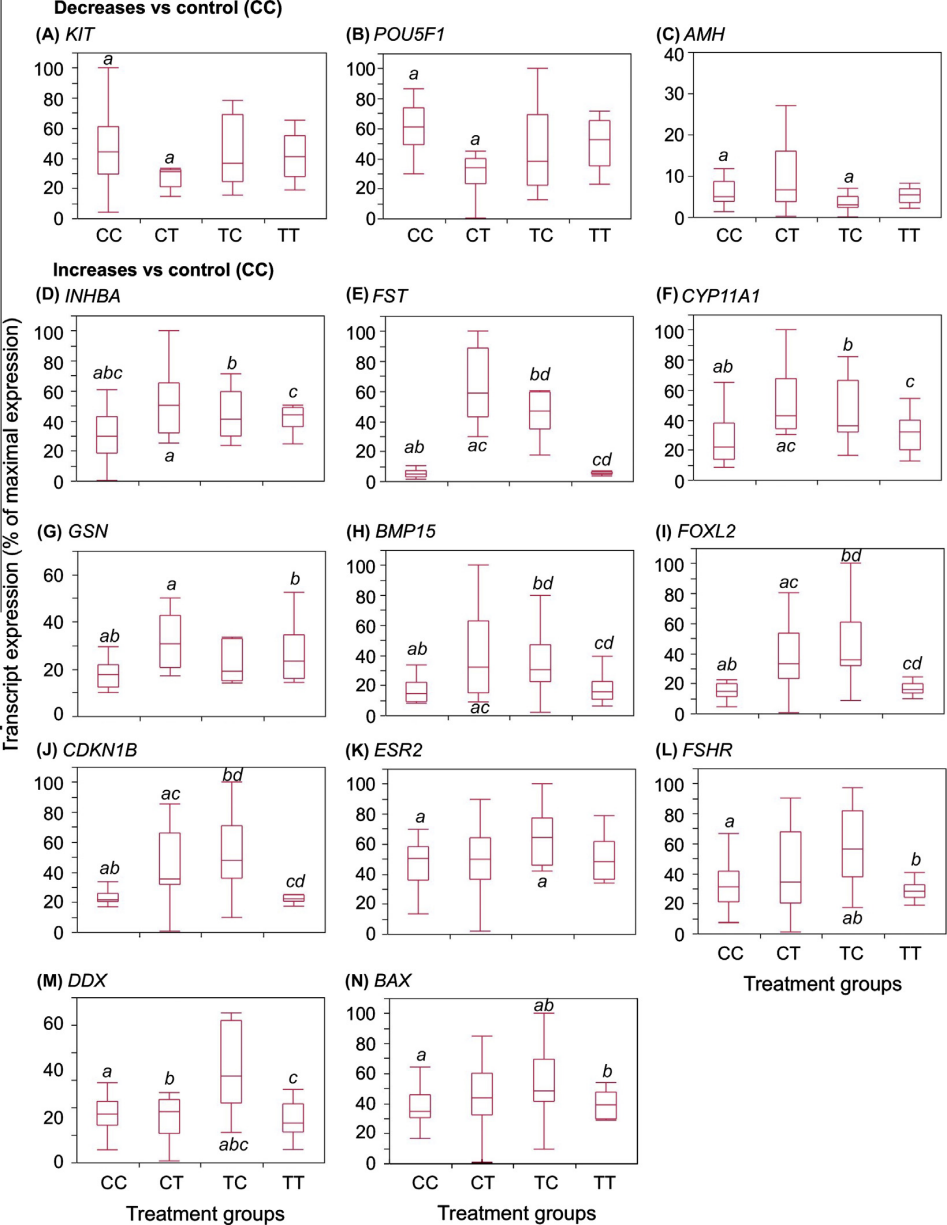
Sewage sludge exposure affects expression of developmental and reproductive mRNA transcripts. (A–C) show transcripts with significant reduction compared with controls while (D–N) show transcript with significant increase in expression relative to controls (CC). Transcript expression was determined by qPCR, normalised relative to the house-keeping gene HPRT1, expressed relative to maximal expression levels for each transcript separately and shown as box and whisker plots. The horizontal line in the boxes show the median values, with the limits of the boxes showing the 25% and 75% quantiles and the whiskers showing the 10% and 90% quantiles. For each transcript separately, common superscripts between groups denote statistically significant differences at *P* < 0.05.

Given the fetal endocrine disturbances and ovarian phenotypic alterations observed in some exposure groups, a gene-candidate expression analysis was conducted for key genes involved in oocyte/follicle formation, differentiation and survival. Of the 23 gene transcripts quantified, 14 exhibited significantly different patterns of expression in ovaries of sewage sludge-exposed fetuses (CT, TC, and TT) relative to controls (CC) (Table 4, Fig. 6). These transcripts fell within functional categories which included: (i) oestrogen production (*CYP11A1*), (ii) germ cell differentiation (*KIT, POU5F1, DDX4*), (iii) cytoskeleton formation (*GSN*), (iv) cell cycle and proliferation/apoptosis (*CDKN1B, BAX*), (v) folliculogenesis (*FOXL2, BMP15, FST, AMH*) and (vi) receptors and signalling factors (*ESR2, FSHR, INHBA*). Assessment of gene expression by qRT-PCR revealed that continuous exposure to sewage sludge (TT) induced fewer alterations than a changing exposure pattern (CT or TC). Only *INHBA* and *GSN* transcripts exhibited differential profiles between CC and TT groups with both being increased in TT fetal ovaries (Table 4, Fig. 6). The greatest incidence of changes in transcript expression was associated with the transfer of ewes from sludge-treated to control pasture at the time of conception (TC). The expression of 10 genes was significantly increased in TC compared to CC ovaries and in 7 genes when compared to TT ovaries (Fig. 6). Genes up-regulated in TC ovaries, relative to those of CC and, to a lesser extent, TT ovaries, included *BAX*, *DDX4, CDKN1B*, *FSHR*, *BMP15*, *FOXL2* and *FST* (Table 4, Fig. 6). However, other follicular and germ cell gene markers were not significantly affected by any exposure (*NOBOX*, *CYP19A1*, *CDC42EP5*, *PTEN*, *MKI67 [KI67]*, *STAR*, *GDF9* and *ESR1*) (Table 4).

The change of exposure from control to sewage sludge-treated pastures at the time of mating (CT) was associated with a different pattern of changes from those associated with the reciprocal cross-over change. Of the gene transcripts investigated, expression was altered in 9 and 7 respectively, relative to that of CC and TT fetuses (Table 4, Fig. 6). Changes in the expression of genes involved in germ cell differentiation (*KIT, POU5F1*) were seen only in the animals that received EC exposure during gestation (CT). For some genes (*INHBA*, *FST CYP11A1*, *GSN*, and B*MP15*), CT exposure represented the most divergent condition relative to the CC group (Fig. 6). Table 5 shows the combined pathway analysis of the transcriptomic and proteomic analyses (Figs. S1–S3). Since the former were based on selected genes known to be important in ovary development and function, the functions identified were not necessarily unexpected. However, the overlap between selected transcripts showing alterations and identified proteins with changed expression indicates fundamental alteration to the ovary of the sewage sludge-exposed fetuses, especially from the CT and TC groups.

### 3.5 Quantification and localisation of gene products in the fetal ovary

**Fig. 7:**
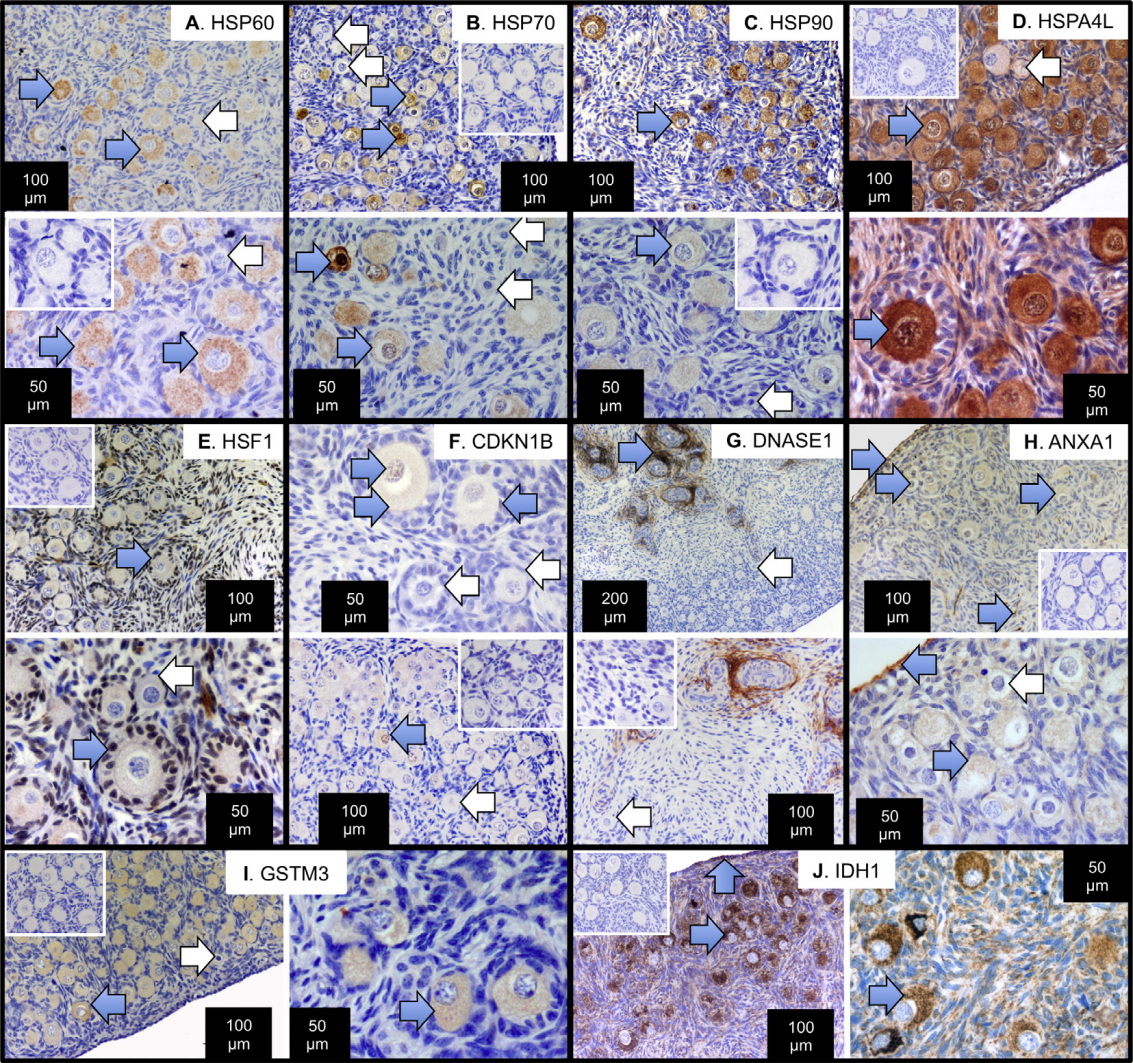
Immunolocalisation of proteins identified as affected by sewage sludge exposure in the fetal sheep ovary. Heat-shock proteins, HSP60 (A), HSP70 (B), HSP90 (C) and HSPA4L (D) were all predominantly oocyte-specific, with strong cytoplasmic staining in most oocytes (blue arrows) but not all oocytes (white arrows). The fifth heat-shock protein, HSF1 (E) was localised in granulosa cells, pre-granulosa cells and many (but not all) somatic cells around the follicles. At higher power, HSF1 expression was detected in the nuclei of granulosa cells (blue arrows), but not in all pre-granulosa cells around primordial or forming primordial follicles (white arrow). CDKN1B (F) was localised in the cytoplasm and/or nuclei of some but not all oocytes and in the nuclei of some granulosa cells (blue arrows). Atretic follicles were CDKN1B-negative, but so were some healthy oocytes and follicles (white arrows). DNASE1 (G) showed intense staining in somatic cells around blood vessels and mesenchymal remnants (blue arrow) although all oocytes were negative (white arrow). ANXA1 (H) exhibited quite wide-spread staining, especially in the ovarian surface epithelium, the cytoplasm of many oocytes and scattered somatic cells (blue arrows). GSTM3 (I) was also principally localised to oocyte cytoplasm (blue arrow) although some oocytes, particularly those showing signs of atresia or dark condensed nuclei were negative (white arrow). IDH1 (J) was localised mainly in oocyte cytoplasm (blue arrow) but also in some somatic cells, including some surface epithelium cells. Positive staining is brown (DAB), counterstained by haematoxylin (blue). The bars denote scale for each image separately. Blue arrows highlight immuno-positive cells and white arrows immuno-negative cells. Two magnifications, taken from different ovaries, are shown for each antigen, separated by a white line. Each antigen is bounded by a black box to simplify interpretation. In all cases IgG-negative slides incubated with non-immune serum of the appropriate species were characterised by an absence of brown stain (one inset panel for each antigen).

Proteins identified in the proteomic analysis altered in sludge exposure groups relative to control were further investigated using WB (Fig. 5) and/or immunohistochemistry in the day 110 gestation fetal ovaries (Fig. 7). HSP90 (spot 75) was oocyte-specific although some oocytes, especially those exhibiting advanced atresia, were immunonegative (Fig. 7C). Results of quantitation of HSP90 were consistent with the outcomes of proteomic measurements: a significant reduction in expression in the sludge-exposed ovaries (Fig. 5A and D). Other members of the heat-shock family, HSP60 and HSP70 were also oocyte-specific (Fig. 7A and B) although only HSP70 showed a quantitative change in protein expression (significant reduction in CT vs CC groups, Fig. 5A, E and F). Two other heat-shock proteins were identified by proteomics, i.e. HSPA4L (spots 45 and 61, Fig. 7D, low expression in many cells but high expression localised in cytoplasm of most, but not all, oocytes) and HSF1 (spot 56, Fig. 7E) predominantly localised in nuclei of granulosa cells and many somatic cells, although not in all pre-granulosa cells surrounding primordial/forming primordial follicles. In the present study, although *GSN* transcript expression was significantly higher in CT and TT groups in relation to controls (Table 4), there was no statistically significant difference in immunoreactive protein (Fig. 5A and C). The cell cycle regulator, CDKN1B, which showed differential transcript expression in the CT and TC groups (Table 4) was expressed in the cytoplasm and/or in the nuclei of some oocytes and a few granulosa cells (Fig. 7F). Oocytes in atretic follicles were CDKN1B-negative. DNASE1 was localised to somatic cells around blood vessels and mesonephric remnants (Fig. 7G) while oocytes and follicles were immunonegative. ANXA1 (Fig. 7H) was quite ubiquitously expressed within the ovary, but more strongly localised to surface epithelium, oocyte cytoplasm and some somatic cells, excluding granulosa cells. The metabolic enzymes GSTM3 (Fig. 7I) and IDH1 (Fig. 7J) were also primarily localised to oocyte cytoplasm, although there was also punctate expression of IDH1 in many somatic cells. Comparison of immunoexpression patterns of these proteins within the fetal ovaries did not exhibit any obvious differences in localisation or in staining intensity (e.g. immunonegative cells in one group vs another), and, therefore, more detailed quantitation was not performed.

## 4 Discussion

We have previously shown that low-level, long-term maternal exposure, to the complex mixtures of environmental chemicals (ECs) present in sewage sludge disrupts fetal ovarian development (Fowler et al., 2008). We now extend these findings, by assessing the relative impacts of pre- and post-conception exposures and show that maternal exposure to sewage sludge is associated with alterations in the fetal ovarian proteome and the transcription of genes crucial for the regulation of folliculogenesis, the primordial follicle and ovary development. The current study demonstrates that the timing of maternal exposure is critical for subsequent effects of EC exposure on the fetal ovary.

### 4.1 Adaptation and/or hormesis?

A surprising finding of the present study is that continuous exposure (TT) did not result in many morphological or gene/protein differences, unlike our previous study (Fowler et al., 2008). The reasons are unclear, but the many uncontrollable variables in the real-world model used; are both a strength (real-life), and a weakness (more variability) of the model. Firstly, while all sewage sludges contain high concentrations of pollutants, including EDCs, their relative proportions differ with each batch of sludge. Secondly, timing of sludge application to pasture and associated climatic conditions differ between the studies while factors such as rainfall and temperature affect volatilisation and bacterial degradation of EDCs. The differential effects observed in the cross-over exposure groups compared to the continuous exposure group may be as a result of changes in pharmacokinetic parameters (Alcorn and McNamara, 2002; Alcorn and McNamara, 2003), which alter maternal and fetal chemical metabolism during pregnancy and development, respectively (Hines, 2008). The absence of significant phenotypic alterations in ovarian histology in continuously-exposed fetuses (TT), while fetuses exposed only post-conception (CT) exhibited significant perturbations, suggests that changes in xenobiotic metabolizing enzyme (DME) expression or activity (e.g.(Daruich et al., 2001)) may have been induced by the pre-mating exposure and then maintained during pregnancy, thereby attenuating the effects of sewage sludge exposure on the fetus. We suggest that the absence of an enhanced EC stimulus to induce protective mechanisms may have raised the sensitivity of the fetus to ECs released as a result of mobilisation of maternal tissues during gestation. This concept, essentially one of hormesis, is increasingly recognised as a possible explanation for observed effects of low doses of environmental chemicals (Calabrese et al., 2008). In addition, there is the issue of individual variability and in our recent study of the effects of pre- and post-natal exposure to sewage sludge 42% of exposed males showed significant testicular abnormalities while 58% did not (Bellingham et al., 2012). The issue of individual variability may be an important factor in the present study where differences between individuals reduced statistical significance (e.g. proportions of MVP +ve vs −ve oocytes). The measurements of the tissue concentration profiles of selected pollutants in the dams and fetuses used in the current study (Rhind et al., 2010b) highlight the complex nature of the factors affecting tissue burdens, their relationship to exposure patterns and the difficulties in elucidating causal relationships between exposure and effect. These complexities, in turn, make it difficult to interpret the observed physiological changes with respect to any single chemical. Finally, we have reported a similar pattern of effects of the different exposures on the thyroid glands from the same fetuses as those used for the present study (Hombach-Klonisch et al., 2013): most pronounced changes were seen in the CT/TC groups.

### 4.2 Ovarian genes/proteins altered in all three sewage sludge exposure groups

The fetal ovaries stemming from mothers exposed to sewage sludge chemicals (before conception, only (TC), after conception, only (CT) or throughout both periods (TT), all exhibited altered expression of members of related pathways (see Table 5), although not necessarily the same members. Five multifunctional genes and proteins highly relevant to ovarian development are common between the three exposure groups. All these factors, known to be sensitive to environmental changes, are involved in response to stress (HSP90AA1, HSPA4L, MVP), regulation of oocyte maturation (INHBA) or apoptosis (DNASE1). Down-regulation of HSP90 in response to chemical exposure has been shown in our (Fowler et al., 2008) and other studies (e.g. Maradonna and Carnevali, 2007; Singh et al., 2009). HSP90 and HSP70 interact with steroid receptors and participate in their translocation to the nucleus (Pratt and Toft, 1997; Pratt and Welsh, 1994), consequently altered expression in response to sewage sludge exposure could affect gene transcription via ovarian steroid receptors. In addition, the down regulation of HSPA4L which is involved in meiosis (Held et al., 2011), could lead to incomplete meiotic prophase I and subsequent apoptotic Type 0 oocytes. Other key ovarian genes altered by exposure have roles in follicle activation, for example MVP (Berger et al., 2009), which was down-regulated in all sludge exposure groups, relative to controls. MVP interacts with the follicular activation inhibitor PTEN and the estrogen receptor (Abbondanza et al., 1998; Yu et al., 2002). Therefore, reduced PTEN function (as opposed to transcript expression which was not altered) associated with a lowered MVP expression, could enable premature activation of primordial follicles and reduction of the follicle pool (Adhikari and Liu, 2009). The increase in MVP expression between early and late gestation supports the suggestion that MVP may play an important role in ovary development and function and it merits further investigation as a marker for disruption of ovarian development.

Other evidence to support an effect of sludge exposure on follicle activation and apoptosis was the increased expression of *INHBA* in all exposed groups. INHBA plays a crucial role in follicle activation (Knight and Glister, 2006; Knight et al., 2011; Lin et al., 2003), thus an increase in intra-ovarian INHBA would be expected to result in an increased rate of follicle activation and subsequent apoptosis. Moreover, expression of DNASE1, which is anti-apoptotic (Mannherz et al., 1995) and involved in the regulation of G-actin polymerization (Weber et al., 1994), was down-regulated, possibly also impairing apoptosis (Napirei et al., 2004). Taken together, alterations in these 5 genes/proteins in all exposure groups highlights several points: (1) maternal exposure to sewage sludge chemicals prior to conception is sufficient to elicit changes in fetal ovarian genes associated with oocyte maturation and apoptosis processes with the potential to dysregulate the ovarian development and its functioning. This is of concern since fetal exposure to sewage sludge chemicals in the TC group was indirect: effects in the TC group were probably attributable to mobilisation of chemicals from maternal fat stores prior to mating, thereby exposing the fetus. (2) Most of the common genes altered across all exposure groups are involved in follicle activation and apoptosis and the direction of gene expression change is consistent with premature follicle activation and increased apoptosis. (3) The genes altered by any exposure to sewage sludge may be expressed in late fetal ovarian development, as demonstrated by gene changes in the TC group where chemical exposure is via maternal mobilisation of ECs. Fat mobilisation increases as gestation progresses and fetal energy demand from the mother increases, thus the fetus is likely to be exposed to constantly-changing levels and mixtures throughout several important windows of development. The sensitivity of the fetus may also change with stage of development. Currently, there is no data concerning toxicokinetics of different environmental chemicals in the fetus, which may be very different from that of adults and this is a concept that requires further investigation. (4) Affected genes belong to pathways involved in stress-responses; which could be activated throughout pregnancy independent of the type of exposure (chronic or acute). Proteome/gene expression analysis on whole-ovary preparations is not without limitations, since the differences in gene expression may be due to shifts in the relative populations of different differentiated cell types. However, taken together the common effects in all exposure groups suggest that exposure to sewage sludge in pre- and post-conception periods has the potential to modify key pathways for fetal ovarian development, which could result in increased follicular apoptosis and reduced follicular growth.

### 4.3 Effects of continuous exposure to sewage sludge

Continuous exposure was clearly associated with altered expression of 3 proteins which were not changed in the crossover exposure groups: HNRNPH1, SEPT11 and IDH1. These genes are involved in apoptosis (Rauch et al., 2010), protein assembly and mitosis, and cellular defence against reactive oxygen species and oxidative stress (Lee et al., 2002) respectively. Therefore, altered expression in the TT group is consistent with the hypothesis that sludge exposure can disrupt fundamental cellular processes in the developing ovary e.g. reducing the ovary’s ability to readily detoxify the reactive intermediates or to repair the resulting damage which contributes to protein and DNA damage in the ovary. Of the genes altered in the TT group, fewer are involved in apoptosis compared to the other exposure groups; therefore it appears that a change in maternal exposure insult may be more damaging for the fetal ovary than continuous maternal exposure in terms of apoptotic effects. This may because the systems for catabolism and detoxification mechanisms which are already in place in the continuous exposure group afford some protection to the fetus, whereas an abrupt change in exposure means that these systems have not been activated and the result may be more damage to the developing ovary. Other than the gene alterations common to all exposure groups, the TT and TC groups alone had no gene alterations in common. On the contrary, the TT and CT groups had 4 genes/transcripts altered in common. In particular, the observed increase in transcription of the gene encoding for gelsolin (GSN) in fetal ovaries from sludge-exposed dams (TT and CT) may be of significance because it is a key regulator of actin filament assembly/disassembly (Kothakota et al., 1997) and apoptosis in the ovary. Although in contrast to our previous study (Fowler et al., 2008), which reported a 2.8-fold reduction in GSN expression in continuously exposed fetuses, the mechanisms by which GSN regulates apoptosis are complex and cell death/proliferation is a fine balance between pro- and anti-apoptotic mechanisms. This highlights the fact that responses may depend on the precise nature and timing of the EDC insult which, in this model is influenced by climatic and management factors. Animals could also be a source of variability e.g. genetic background and differences in maternal and/or fetal metabolism. One consequence of the “real-life” model we employed is that it is not possible to control all variables, which is quite different from a more typical laboratory rodent chemical exposure study. However, although the latter allows more mechanistic insights, our study design enables more realistic assessment of likely effects of chemical exposures on our own species, which lives in highly variable and uncontrolled environments. Both study design types are essential to properly understand risks from chemical exposures.

### 4.4 Common and differential effects of pre- and post- conception exposures

Six of the 13 fetal ovarian genes/proteins which showed increased expression in both the CT and TC groups (relative to the CC group, Tables 3 and 4) have crucial roles in ovarian development and have been implicated in POF and PCOS, indicating possible mechanisms of EC action. For example FOXL2, an essential transcription factor in ovarian development, and follistatin (FST) with which FOXL2 interacts, together with estrogen receptor beta (*ESR2)* (Kashimada et al., 2011; Schmidt et al., 2004), were both up regulated in TC and CT groups. FOXL2 is strongly increased during primary-secondary follicle transition in sheep (Jagarlamudi and Rajkovic, 2012; Kuo et al., 2011; Uhlenhaut and Treier, 2011) and so the up-regulation of *FST i*n the CT and TC fetal ovaries could impair ovarian follicle development and increase intra-ovarian androgen production. In other animal models, such increased expression of *FST* results in a PCOS-like disruption of the ovary (Guo et al., 1998). Increased *CYP11A1* mRNA expression, as observed in the current study, has also been proposed as a factor in PCOS (Nelson et al., 1999) while increased *CDKN1B* (P27KIP1) expression, which maintains the dormancy of primordial follicles (Hirashima et al., 2011; Rajareddy et al., 2007) may contribute to increased follicle atresia after early follicle recruitment. Examination of follicle type and number revealed that for most follicle types (Type 0, type 1 and type 2), there was no significant difference between any of the exposure groups despite changes in expression of key ovarian genes across all exposure groups. However, there was a reduced proportion of activated follicles (type 1a) in post-conception maternal exposure (CT) fetal ovaries which were also associated with a higher (although not significant) density of remaining naked oocytes (type 0) and the greatest number of gene alterations compared to the other exposure groups. The discrepancy between altered gene expression yet absent effects on follicle type in some exposure groups highlight the limitations of using follicle counts as the only predictor of ‘normal’ ovarian development. In spite of an almost normal morphology, key pathways for follicle differentiation can be disturbed and the effects of these disturbances on fertility could be visible only several years after. The developmental stage when the effects are measured is also a source of variation in the reported studies and could explain contradictory effects reported in the literature.

In CT fetal ovaries, the increased incidence of unhealthy oocytes observed may be a function of the reduced expression of two germ cell specific genes, *KIT* and *POU5F1* (OCT3/4). Interestingly, the expression of the pro-survival gene KIT is decreased when human fetal ovaries were exposed *in vitro* to high doses of Dexamethasone. This chemical impairs human fetal oogenesis through an increase in apoptosis (Poulain et al., 2012). POU5F1 plays a pivotal role in maintaining the pluripotency and germline potential and self-renewal of primordial germ cells (Stoop et al., 2005). The reduction in POU5F1 expression observed in the present study could impair differentiation and survival of oogonia. The TC fetal ovaries showed increased expression of *DDX4* (VASA) involved in assembly and transport of mRNAs during oogenesis (Castrillon et al., 2000) and *BAX* genes, which play a central role in follicular atresia by determining germ cell loss in the developing mammalian ovary (De Felici et al., 2005; Greenfeld et al., 2007) and are involved in POF. In other studies involving disruptive ECs, an increase in oocyte death was similarly associated with increased BAX expression (Matikainen et al., 2001, 2002). Increased expression of *DDX4* and *BAX* in the TC ovaries is consistent with early activation of follicles and this could result in a reduced follicle pool, early depletion of follicles and compromised adult reproductive function. Changes in structural and cytoskeleton proteins were also identified in fetuses from crossover ewes. LMNA (Lamin A/C), a deficiency of which is associated with infertility in women (Vantyghem et al., 2008) was down-regulated in the CT and TC groups while VIM, a mesenchymal intermediate filament was up-regulated in the CT group. Collectively, these changes are likely to induce enhanced apoptosis and other perturbations in fundamental cellular processes (e.g. cell division) in the fetal ovary and such effects are reflected in the involvement of comparable changes in their expression in the development of various cancers (Capo-chichi et al., 2011; Gonzalez-Suarez et al., 2009). ANXA1 is down-regulated in breast cancer and has protective roles against the proliferative action of estrogens in cancer cells (Castro-Caldas et al., 2001; Huggins et al., 2009; Perretti and D’Acquisto, 2009). Therefore, the down-regulation of ANXA1 (widely expressed in the fetal ovary, Fig. 7), in the cross-over groups (CT and TC) is consistent with increased cell proliferation and apoptosis and probably associated with dysregulation of primordial follicle formation/progression and increased proportion of unhealthy/atretic oocytes in the CT exposure group.

## 5 Conclusions

The change in EDC exposure (via sludge) at mating, resulted in significant changes, relative to unexposed controls, in fetal ovarian histology, with associated changes in the transcription of genes critical for ovarian development. Some of the genes and proteins dysregulated by sewage sludge exposure have been implicated in ovarian pathologies in humans, suggesting that low-level exposure to multiple ECs such as are found in sewage sludge, may contribute to the aetiology of these diseases. This study also shown that morphological criteria, such as follicle counting, are not, alone, sufficient to assess the disturbances occurring during ovarian development. Complementary studies associating transcriptome and proteome analysis are needed for a functional assessment of the ovarian differentiation. Some of the subtle changes observed during fetal life may become visible only several years later. We conclude that changes in environmental chemical exposure both before and after conception can differentially perturb ovarian development of offspring and have the potential to adversely affect ovarian function in adulthood. The period preceding the gestation must be crucial in term of accumulation or salting out of EDCs. Particular attention to this period may be important for women planning conception and raises the question of slimming diets or weight loss surgery during just before conception. Our findings also provide valuable insight into some of the variable data on human exposures and phenotypic/genotypic consequences.

## Figures and Tables

**Table 1:** Effects of chemical cocktails in sewage sludge on day 110 female fetus: morphological, ovarian and endocrine characteristics. Values are mean ± SEM. Common superscripts denote differences at *P* < 0.05. Paired organs are combined into a single weight. Group numbers are shown in parentheses with group codes. Values are follicle number/mm^2^.

Treatment groups	Continuous exposure profile		Cross-over exposure profile	
	CC (15)	TT (12)	CT (8)	TC (7)
*Morphology*				
Body weight (g)	1845 ± 50	1829 ± 84	1922 ± 84	1827 ± 73
Uterus (mg)	436 ± 31	431 ± 26	368 ± 49	363 ± 52

*Endocrinology*				
LH (ng/ml)	1.65 ± 0.33	1.79 ± 0.37	1.73 ± 0.75	2.49 ± 0.92
FSH (ng/ml)	1.28 ± 0.08	1.52 ± 0.11^a^	1.18 ± 0.13^a^	1.37 ± 0.16
Testosterone (ng/ml)	0.11 ± 0.01	0.11 ± 0.01	0.12 ± 0.01	0.12 ± 0.01
Estradiol (pg/ml)	5.7 ± 2.4	1.5 ± 0.9^a^	7.6 ± 4.4^a^	4.0 ± 1.5
Progesterone (nmol/l)	63.6 ± 8.0	81.4 ± 17.2	72.3 ± 10.7	47.1 ± 6.1
Inhibin A (pg/ml)	15.2 ± 0.6^a^	17.3 ± 0.8^a^	16.5 ± 0.9	16.9 ± 0.8

*Ovarian characteristics*				
Ovaries (mg)	41 ± 3^a^	56 ± 7^a^	55 ± 7	50 ± 4
Ovaries assessed by microscopy	7	5	4	7
Total Oocyte/Follicle density	109 ± 15	109 ± 8	98 ± 26	116 ± 11
Oocyte/Follicle density^a^				
Type 0	2 ± 1	1 ± 1	4 ± 1	1 ± 1
Type 1	49 ± 11	52 ± 7	68 ± 23	68 ± 13
Type 1a	51 ± 7^a^	50 ± 5^b^	21 ± 6^ab^	38 ± 4
Type 2	7 ± 1	6 ± 1	5 ± 2	9 ± 3

a Type 0: isolated oocytes; type 1: primordial follicles; type 1a: transitory/activated follicle; type 2: primary follicle (1-<2 complete layers of granulosa cells; type 3 onwards; insufficient numbers for robust statistical analysis.

**Table 2:** Numbers of ovarian protein spots (in 2-DE gels) with significantly different spot volumes following differential maternal exposures to chemical cocktails in sewage sludge fertiliser.

Treatment groups	Continuous exposure profile		Cross-over exposure profile	
	CC	TT	CT	TC
CC		42	53	42
TT	42		36	31
CT	53	36		39
TC	42	31	39	

**Table 3:** Fetal ovarian proteins exhibiting differential expression following continuous or cross-over exposure to chemical cocktails in sewage sludge fertiliser. The accession number is derived from NCBI. Fold change relative to the normalised spot volumes for the CC group are increased if marked “+” and decreased if marked “−“. Fold-change values attaining statistical significance are highlighted in bold. *P* values are derived by post hoc tests of log-normalised spot volumes. Spots containing significant protein matches that cannot be discriminated between are marked with * next to the spot number. Where the same protein is identified in different spots vertical lines join the spot numbers.

Spot #	Gene symbol	Protein name *Relevant function*	MW (kDa)	pI	MOWSE score	Accession number (NCBI)	Fold-change and (*P* value) vs CC		
							TT	CT	TC
*Heat-shock/stress responses*									
45	*HSPA4L*	Heat shock 70 kDa protein 4L	95	5.6	419	XP533297	−1.23 (0.022)	−1.27 (0.010)	−1.53 (<0.001)
61		*Chaperone activity in stress responses*			538		−1.14 (0.152)	−1.29 (0.009)	−1.39 (0.002)
56^*^	*HSF1*	Heat shock factor protein 1 *binds heat shock promoter elements (HSE) and activates transcription*	57	5.4	763	BAD12572	+1.38 (0.015)	+1.36 (0.014)	−1.04 (0.758)
75	*HSP90AA1*	Heat shock protein HSP 90-alpha	85	4.9	1564	NP999138	−1.28 (0.004)	−1.18 (0.036	−1.15 (0.064)
		*Multiple roles as molecular chaperone, including stress response, cell cycle protein folding, steroid receptor activity*							

*mRNA processing*									
79^*^	*HNRNPH3*								
		Heterogeneous nuclear ribonucleoprotein H3	37	6.4	324	NP036339	−1.04 (0.431)	+1.19 (0.007)	+1.06 (0.286)
		*Nuclear mRNA splicing, via spliceosome*							
84^*^	*HNRNPH1*	Heterogeneous nuclear ribonucleoprotein H1	50	5.9	599	NP005511	+1.19 (0.007)	−1.00 (0.958	−1.01 (0.890)
		*Nuclear mRNA splicing, via spliceosome*							

*Structural component/apoptosis*									
44	*VIM*	Vimentin	54	5.0	1432	ABP48145	+1.19 (0.239)	+1.45 (0.020)	−1.07 (0.684
		*Class-III intermediate filaments in non-epithelial cells, cellular component disassembly involved in apoptosis*							
66	*KRT1*^a^	Keratin, type II cytoskeletal 1	66	8.2	216	AAG41947	−1.19 (0.055)	+1.11 (0.178)	+1.09 (0.226)
		*May regulate the activity of kinases via binding to integrin beta-1 (ITB1) and RACK1/GNB2L1*							
86	*LMNA*	Lamin A/C	75	6.6	1064	XP864434	−1.05 (0.232)	−1.18 (0.002)	−1.15 (0.004)
		*Component of the nuclear lamina, role in nuclear assembly, chromatin organization, nuclear membrane and telomere dynamics*							
42	*DNASE1*	Deoxyribonuclease-1	29	5.1	503	1DKNA	−1.39 (0.003)	−1.58 (<0.001)	−1.48 (0.001)
		*Binds specifically to G-actin and blocks actin polymerization, involved in processes including apoptosis*							

*Signal transduction/cell cycle*									
47	*MVP*	Major vault protein	99	5.5	2036	NP001030394	−1.52 (0.003)	−1.23 (0.089)	−1.41 (0.014)
67		*Required for normal vault structure: Vaults are multi-subunit structures acting as scaffolds for proteins involved in signal transduction*			1599		−1.33 (0.001)	−1.26 (0.003)	−1.33 (0.001)
78	*FGB*	Fibrinogen beta chain	53	6.3	581	AAI49542	+1.09 (0.240)	+1.26 (0.008)	+1.18 (0.044)
		*Protein polymerization and signal transduction*							
84^*^	*SEPT11*	Septin 11	50	6.4	622	NP060713	+1.19 (0.007)	−1.00 (0.958)	−1.01 (0.890)
		*Filament-forming cytoskeletal GTPase with roles in cell cycle*							
82	*PSMA4*	Proteasome subunit alpha type-4	26	7.8	444	CAA62960	+1.07 (0.214	+1.21 (0.004)	+1.15 (0.020)
		*DNA damage response, signal transduction by p53 class mediator resulting in cell cycle arrest*							

*Enzyme activity/detoxification*									
76	*DLD*	Dihydrolipoyl dehydrogenase, mitochondrial	51	6.4	509	1ZMCA	−1.09 (0.339)	−1.23 (0.033)	−1.27 (0.013)
		*A component of the glycine cleavage system and alpha-ketoacid dehydrogenase complexes*							
65	*IDH1*	Isocitrate dehydrogenase [NADP] cytoplasmic	47	6.3	933	NP001009276	−1.28 (0.003)	−1.11(0.155)	+1.04 (0.472)
		*Participates in glyoxylate bypass and tricarboxylic acid cycle*							
21	*GSTM3*	Glutathione S-transferase Mu 3	27	6.7	183	XP537037	+1.05 (0.770)	−1.77 (0.001)	+1.07 (0.674)
		*Conjugation of reduced glutathione to a wide number of exogenous and endogenous hydrophobic electrophiles*							
79^*^	*AKR7A2*	Aflatoxin B1 aldehyde reductase member 2	40	6.7	352	AAI49541	−1.04 (0.431)	+1.19 (0.007)	+1.06 (0.286)
		*Catalyzes the NADPH-dependent reduction of succinic semialdehyde to gamma-hydroxybutyrate*							
62^*^	*AKR1B1*	Aldose reductase	36	5.8	773	P16116	+1.02	−1.35	−1.30
		*C21-steroid hormone biosynthetic process*					(0.759)	(0.002)	(0.005)
87	*GSTM1*	Glutathione S-transferase Mu 1	26	6.9	378	NP787019	+1.04 (0.431)	+1.16 (0.008)	+1.12 (0.031)
		*Xenobiotic metabolic processes*							

*Molecular binding/transport*									
56^*^	*AFP*	Alpha-fetoprotein	70	5.9	594	NP001029434	+1.38 (0.015)	+1.36 (0.014)	−1.04 (0.758)
60		*Binds copper, nickel, and fatty acids, less than 2% of human AFP binds estrogens*			896		+1.12 (0.217)	+1.39 (0.003)	+1.05 (0.610)
83					939		+1.09 (0.138)	+1.20 (0.005)	+1.03 (0.665)
70	*TF*^a^	Serotransferrin	80	6.9	824	ACJ03828	−1.14 (0.072)	+1.06 (0.461)	+1.15 (0.067)
		*Iron binding/transport*							
62^*^	*ANXA1*	Annexin A1	39	6.4	268	CAA39971	+1.02 (0.759)	−1.35 (0.002)	−1.30 (0.005)
		*Calcium/phospholipid-binding protein, promotes membrane fusion, is involved in exocytosis and is anti-apoptotic*							

a CT and TC significantly different to TT.

**Table 4:** Summary of exposure effects on fetal ovarian transcripts. See Fig. 3.

Significantly (*P* < 0.05) altered compared with control (CC)			Not significantly affected by exposures
CT	TC	TT	
Decreased			*NOBOX*
*KIT*	*AMH*		*CYP19A1*
*POU5F1*			*CDC42EP5*
*Increased*			*PTEN*
*INHBA*	*INHBA*	*INHBA*	*MKI67*
*CYP11A1*	*CYP11A1*		*STAR*
*BMP15*	*BMP15*		*GDF9*
*GSN*		*GSN*	*ESR1*
*FST*	*FST*		
*FOXL2*	*FOXL2*		
*CDKN1B*	*CDKN1B*		
	*ESR2*		
	*DDX4*		
	*FSHR*		
	*BAX*		

**Table 5:** Networks affected by sewage sludge exposure. Genes shown in bold are those, or their products, identified as significantly altered in at least one treatment group compared with controls (CC) in the present study. Analysis was performed using IPA.

ID	Molecules in network	Score	Focus molecules	Top functions
1	**AKR1B1**, **AMH**, AMPK, **ANXA1**, Ap1, **BAX**, **BMP15**, caspase, **CDKN1B**, Cg, Creb, Cyclin A, **CYP11A1**, ERK1/2, **ESR2**, estrogen receptor, **FOXL2**, FSH, **FSHR**, **FST**, Growth hormone, **GSTM1**, **GSTM3**, Hsp27, IL1, I**NHA**, Inhibin, **KIT**, LDL, Lh, **LMNA**, Mek, **MVP**, TF, TSH	46	18	Renal and Urological System Development and Function, Reproductive System Development and Function, Embryonic Development
2	14–3-3, 26s Proteasome, **AFP**, **AKR7A2**, Akt, Alpha catenin, calpain, CD3, **DDX4**, ERK, **FGB**, Histone h3, **HSF1** (includes EG:15499), Hsp70, Hsp90, HSP, **HSP90AA1**, **HSPA4L**, **IDH1**, Insulin, Interferon alpha, Jnk, **KRT1**, Mapk, NFkB (complex), P38 MAPK, PDGF BB, PI3 K (complex), Pka, Pkc(s), **POU5F1**, Tgf beta, Ubiquitin, Vegf, **VIM**	25	11	Post-Translational Modification, Protein Folding, Organismal Injury and Abnormalities
3	ATL3, BRAT1, C12orf5, C2orf29, CCDC8, CHIC1, CISD1, **DLD**, **DNASE1**, FAM120A, FIGNL1, GPD1L, HECW2, **HNRNPH1**, **HNRNPH3,** MAGEB1, MIS18A, MOCOS, MRPL46, OGDHL, PADI2, PROSC, **PSMA4**, PSMG2, RBM34, SCPEP1, **SEPT11**, SH3BGRL2, THG1L, TP53 (includes EG:22059), TPRKB, TTLL5, UBC, UBL3, ZNF84	12	6	Cell Morphology, Cellular Compromise, DNA Replication, Recombination, and Repair

## References

[b0005] Abbondanza C., Rossi V., Roscigno A., Gallo L., Belsito A., Piluso G., Medici N., Nigro V., Molinari A.M., Moncharmont B., Puca G.A. (1998). Interaction of vault particles with estrogen receptor in the MCF-7 breast cancer cell. J. Cell Biol..

[b0010] Adhikari D., Liu K. (2009). Molecular mechanisms underlying the activation of mammalian primordial follicles. Endocr. Rev..

[b0015] Alcorn J., McNamara P.J. (2002). Ontogeny of hepatic and renal systemic clearance pathways in infants: Part II. Clin. Pharmacokinet..

[b0020] Alcorn J., McNamara P.J. (2003). Pharmacokinetics in the newborn. Adv. Drug Deliv. Rev..

[b0025] Bellingham M., Fowler P.A., Amezaga M.R., Rhind S.M., Cotinot C., Mandon-Pepin B., Sharpe R.M., Evans N.P. (2009). Exposure to a complex cocktail of environmental endocrine-disrupting compounds disturbs the kisspeptin/GPR54 system in ovine hypothalamus and pituitary gland. Environ. Health Perspect..

[b0030] Bellingham M., Fowler P.A., Amezaga M.R., Whitelaw C.M., Rhind S.M., Cotinot C., Mandon-Pepin B., Sharpe R.M., Evans N.P. (2010). Foetal hypothalamic and pituitary expression of gonadotrophin-releasing hormone and galanin systems is disturbed by exposure to sewage sludge chemicals via maternal ingestion. J. Neuroendocrinol..

[b0035] Bellingham M., McKinnell C., Fowler P.A., Amezaga M.R., Zhang Z., Rhind S.M., Cotinot C., Mandon-Pepin B., Evans N.P., Sharpe R.M. (2012). Foetal and post-natal exposure of sheep to sewage sludge chemicals disrupts sperm production in adulthood in a subset of animals. Int. J. Androl..

[b0040] Berger W., Steiner E., Grusch M., Elbling L., Micksche M. (2009). Vaults and the major vault protein: novel roles in signal pathway regulation and immunity. Cell. Mol. Life Sci..

[b0045] Bigsby R.M., Caperell-Grant A., Madhukar B.V. (1997). Xenobiotics released from fat during fasting produce estrogenic effects in ovariectomized mice. Cancer Res..

[b0050] Calabrese E.J., Stanek E.J., Nascarella M.A., Hoffmann G.R. (2008). Hormesis predicts low-dose responses better than threshold models. Int. J. Toxicol..

[b0055] Capo-chichi C.D., Cai K.Q., Simpkins F., Ganjei-Azar P., Godwin A.K., Xu X.X. (2011). Nuclear envelope structural defects cause chromosomal numerical instability and aneuploidy in ovarian cancer. BMC Med..

[b0060] Castrillon D.H., Quade B.J., Wang T.Y., Quigley C.A., Crum C.P. (2000). The human VASA gene is specifically expressed in the germ cell lineage. Proc. Natl. Acad. Sci. USA.

[b0065] Castro-Caldas M., Duarte C.B., Carvalho A.R., Lopes M.C. (2001). 17Beta-estradiol promotes the synthesis and the secretion of annexin I in the CCRF-CEM human cell line. Mediators Inflamm..

[b0070] Colborn T., vom Saal F.S., Soto A.M. (1993). Developmental effects of endocrine-disrupting chemicals in wildlife and humans. Environ. Health Perspect..

[b0075] Craig Z.R., Wang W., Flaws J.A. (2011). Endocrine-disrupting chemicals in ovarian function: effects on steroidogenesis, metabolism and nuclear receptor signaling. Reproduction.

[b0080] Daruich J., Zirulnik F., Gimenez M.S. (2001). Effect of the herbicide glyphosate on enzymatic activity in pregnant rats and their fetuses. Environ. Res..

[b0085] De Felici M., Klinger F.G., Farini D., Scaldaferri M.L., Iona S., Lobascio M. (2005). Establishment of oocyte population in the fetal ovary: primordial germ cell proliferation and oocyte programmed cell death. Reprod. Biomed. Online.

[b0090] Evans N.P., Dahl G.E., Glover B.H., Karsch F.J. (1994). Central regulation of pulsatile gonadotropin-releasing hormone (GnRH) secretion by estradiol during the period leading up to the preovulatory GnRH surge in the ewe. Endocrinology.

[b0095] Fowler P.A., Dora N.J., McFerran H., Amezaga M.R., Miller D.W., Lea R.G., Cash P., McNeilly A.S., Evans N.P., Cotinot C., Sharpe R.M., Rhind S.M. (2008). In utero exposure to low doses of environmental pollutants disrupts fetal ovarian development in sheep. Mol. Hum. Reprod..

[b0100] Fowler P.A., Flannigan S., Mathers A., Gillanders K., Lea R.G., Wood M.J., Maheshwari A., Bhattacharya S., Collie-Duguid E.S., Baker P.J., Monteiro A., O’Shaughnessy P.J. (2009). Gene expression analysis of human fetal ovarian primordial follicle formation. J. Clin. Endocrinol. Metabol..

[b0105] Fowler P.A., Bellingham M., Sinclair K.D., Evans N.P., Pocar P., Fischer B., Schaedlich K., Schmidt J.S., Amezaga M.R., Bhattacharya S., Rhind S.M., O’Shaughnessy P.J. (2012). Impact of endocrine-disrupting compounds (EDCs) on female reproductive health. Mol. Cell. Endocrinol..

[b0110] Gonzalez-Suarez I., Redwood A.B., Gonzalo S. (2009). Loss of A-type lamins and genomic instability. Cell Cycle.

[b0115] Gray L.E., Ostby J., Furr J., Price M., Veeramachaneni D.N., Parks L. (2000). Perinatal exposure to the phthalates DEHP, BBP, and DINP, but not DEP, DMP, or DOTP, alters sexual differentiation of the male rat. Toxicol. Sci..

[b0120] Greenfeld C.R., Pepling M.E., Babus J.K., Furth P.A., Flaws J.A. (2007). BAX regulates follicular endowment in mice. Reproduction.

[b0125] Guillette L.J., Gross T.S., Masson G.R., Matter J.M., Percival H.F., Woodward A.R. (1994). Developmental abnormalities of the gonad and abnormal sex hormone concentrations in juvenile alligators from contaminated and control lakes in Florida. Environ. Health Perspect..

[b0130] Guo Q., Kumar T.R., Woodruff T., Hadsell L.A., DeMayo F.J., Matzuk M.M. (1998). Overexpression of mouse follistatin causes reproductive defects in transgenic mice. Mole. Endocrinol..

[b0135] Held T., Barakat A.Z., Mohamed B.A., Paprotta I., Meinhardt A., Engel W., Adham I.M. (2011). Heat-shock protein HSPA4 is required for progression of spermatogenesis. Reproduction.

[b0140] Herreros M.A., Gonzalez-Bulnes A., Inigo-Nunez S., Letelier C., Contreras-Solis I., Ros-Rodriguez J.M., Encinas T. (2010). Pregnancy-associated changes in plasma concentration of the endocrine disruptor di(2-ethylhexyl) phthalate in a sheep model. Theriogenology.

[b0145] Hines R.N. (2008). The ontogeny of drug metabolism enzymes and implications for adverse drug events. Pharmacol. Ther..

[b0150] Hirashima Y., Moniruzzaman M., Miyano T. (2011). P27(Kip1) negatively regulates the activation of murine primordial oocytes. J. Reprod. Develop..

[b0155] Hombach-Klonisch S., Danescu A., Begum F., Amezaga M.R., Rhind S.M., Sharpe R.M., Evans N.P., Bellingham M., Cotinot C., Mandon-Pepin B., Fowler P.A., Klonisch T. (2013). Peri-conceptional changes in maternal exposure to sewage sludge chemicals disturbs fetal thyroid gland development in sheep. Mol. Cell. Endocrinol..

[b0160] Huggins A., Paschalidis N., Flower R.J., Perretti M., D’Acquisto F. (2009). Annexin-1-deficient dendritic cells acquire a mature phenotype during differentiation. FASEB J.: Official Publ. Federat. Am. Soc. Exper. Biol..

[b0165] Jagarlamudi K., Rajkovic A. (2012). Oogenesis: transcriptional regulators and mouse models. Mol. Cell. Endocrinol..

[b0170] Kashimada K., Pelosi E., Chen H., Schlessinger D., Wilhelm D., Koopman P. (2011). FOXL2 and BMP2 act cooperatively to regulate follistatin gene expression during ovarian development. Endocrinology.

[b0175] Knight P.G., Glister C. (2006). TGF-beta superfamily members and ovarian follicle development. Reproduction.

[b0180] Knight P.G., Feist S.A., Tannetta D.S., Bleach E.C., Fowler P.A., O’Brien M., Groome N.P. (1998). Measurement of inhibin-A (alpha beta A dimer) during the oestrous cycle, after manipulation of ovarian activity and during pregnancy in ewes. J. Reprod. Fertil..

[b0185] Knight P.G., Satchell L., Glister C. (2011). Intra-ovarian roles of activins and inhibins. Mol. Cell. Endocrinol..

[b0190] Kothakota S., Azuma T., Reinhard C., Klippel A., Tang J., Chu K., McGarry T.J., Kirschner M.W., Koths K., Kwiatkowski D.J., Williams L.T. (1997). Caspase-3-generated fragment of gelsolin: effector of morphological change in apoptosis. Science.

[b0195] Kuo F.-T., Bentsi-Barnes I.K., Barlow G.M., Pisarska M.D. (2011). Mutant Forkhead L2 (FOXL2) proteins associated with premature ovarian failure (POF) dimerize with wild-type FOXL2, leading to altered regulation of genes associated with granulosa cell differentiation. Endocrinology.

[b0200] Lee S.M., Koh H.-J., Park D.-C., Song B.J., Huh T.-L., Park J.-W. (2002). Cytosolic NADP+-dependent isocitrate dehydrogenase status modulates oxidative damage to cells. Free Radical Biol. Med..

[b0205] Lin S.Y., Morrison J.R., Phillips D.J., de Kretser D.M. (2003). Regulation of ovarian function by the TGF-beta superfamily and follistatin. Reproduction.

[b0210] Lundy T., Smith P., O’Connell A., Hudson N.L., McNatty K.P. (1999). Populations of granulosa cells in small follicles of the sheep ovary. J. Reprod. Fertil..

[b0215] Magnusson U. (2012). Environmental endocrine disruptors in farm animal reproduction: research and reality. Reprod. Domest. Anim..

[b0220] Mandon-Pepin B., Oustry-Vaiman A., Vigier B., Piumi F., Cribiu E., Cotinot C. (2003). Expression profiles and chromosomal localization of genes controlling meiosis and follicular development in the sheep ovary. Biol. Reprod..

[b0225] Mannherz H.G., Peitsch M.C., Zanotti S., Paddenberg R., Polzar B. (1995). A new function for an old enzyme: the role of DNase I in apoptosis. Curr. Top. Microbiol. Immunol..

[b0230] Maradonna F., Carnevali O. (2007). Vitellogenin, zona radiata protein, cathepsin D and heat shock protein 70 as biomarkers of exposure to xenobiotics. Biomarkers: Biochem. Indicat. Expo., Response, Suscept. Chem..

[b0235] Matikainen T., Perez G.I., Jurisicova A., Pru J.K., Schlezinger J.J., Ryu H.Y., Laine J., Sakai T., Korsmeyer S.J., Casper R.F., Sherr D.H., Tilly J.L. (2001). Aromatic hydrocarbon receptor-driven Bax gene expression is required for premature ovarian failure caused by biohazardous environmental chemicals. Nat. Genet..

[b0240] Matikainen T.M., Moriyama T., Morita Y., Perez G.I., Korsmeyer S.J., Sherr D.H., Tilly J.L. (2002). Ligand activation of the aromatic hydrocarbon receptor transcription factor drives Bax-dependent apoptosis in developing fetal ovarian germ cells. Endocrinology.

[b0245] McNatty K.P., Smith P., Hudson N.L., Heath D.A., Tisdall D.J., O W.S., Braw-Tal R. (1995). Development of the sheep ovary during fetal and early neonatal life and the effect of fecundity genes. J. Reprod. Fert..

[b0250] McNeilly A.S., Jonassen J.A., Fraser H.M. (1986). Suppression of follicular development after chronic LHRH immunoneutralization in the ewe. J. Reprod. Fertil..

[b0255] Meerts I.A., Letcher R.J., Hoving S., Marsh G., Bergman A., Lemmen J.G., van der Burg B., Brouwer A. (2001). In vitro estrogenicity of polybrominated diphenyl ethers, hydroxylated PDBEs, and polybrominated bisphenol A compounds. Environ. Health Perspect..

[b0260] Napirei M., Wulf S., Mannherz H.G. (2004). Chromatin breakdown during necrosis by serum Dnase1 and the plasminogen system. Arthritis Rheum..

[b0265] Nelson V.L., Legro R.S., Strauss J.F., McAllister J.M. (1999). Augmented androgen production is a stable steroidogenic phenotype of propagated theca cells from polycystic ovaries. Mole. Endocrinol. (Baltimore, Md.).

[b0270] O’Shaughnessy P.J., Monteiro A., Bhattacharya S., Fowler P.A. (2011). Maternal smoking and fetal sex significantly affect metabolic enzyme expression in the human fetal liver. J. Clin. Endocrinol. Metab..

[b0275] Paul C., Rhind S.M., Kyle C.E., Scott H., McKinnell C., Sharpe R.M. (2005). Cellular and hormonal disruption of fetal testis development in sheep reared on pasture treated with sewage sludge. Environ. Health Perspect..

[b0280] Perretti M., D’Acquisto F. (2009). Annexin A1 and glucocorticoids as effectors of the resolution of inflammation, Nature reviews. Immunology.

[b0285] Poulain M., Frydman N., Duquenne C., N’Tumba-Byn T., Benachi A., Habert R., Rouiller-Fabre V., Livera G. (2012). Dexamethasone induces germ cell apoptosis in the human fetal ovary. J. Clin. Endocrinol. Metab..

[b0290] Pratt W.B., Toft D.O. (1997). Steroid receptor interactions with heat shock protein and immunophilin chaperones. Endocr. Rev..

[b0295] Pratt W.B., Welsh M.J. (1994). Chaperone functions of the heat shock proteins associated with steroid receptors. Semin. Cell Biol..

[b0300] Rajareddy S., Reddy P., Du C., Liu L., Jagarlamudi K., Tang W., Shen Y., Berthet C., Peng S.L., Kaldis P., Liu K. (2007). P27kip1 (cyclin-dependent kinase inhibitor 1B) controls ovarian development by suppressing follicle endowment and activation and promoting follicle atresia in mice. Mol. Endocrinol..

[b0305] Rauch J., O’Neill E., Mack B., Matthias C., Munz M., Kolch W., Gires O. (2010). Heterogeneous nuclear ribonucleoprotein H blocks MST2-mediated apoptosis in cancer cells by regulating A-Raf transcription. Cancer Res..

[b0310] Rhind S.M. (2002). Endocrine disrupting compounds and farm animals: their properties, actions and routes of exposure. Domest. Anim. Endocrinol..

[b0400] Rhind S.M., Kyle C.E., Telfer G., Duff E.L., Smith A. (2005). Alkyl phenols and diethylhexyl phthalate in tissues of sheep grazing pastures fertilized with sewage sludge or inorganic fertilizer. Environ. Health. Perspect..

[b0320] Rhind S.M. (2005). Are endocrine disrupting compounds a threat to farm animal health, welfare and productivity?. Reprod. Domest. Anim..

[b0325] Rhind S.M., Kyle C.E., Mackie C., McDonald L. (2009). Accumulation of endocrine disrupting compounds in sheep fetal and maternal liver tissue following exposure to pastures treated with sewage sludge. J. Environ. Monit..

[b0330] Rhind S.M., Evans N.P., Bellingham M., Sharpe R.M., Cotinot C., Mandon-Pepin B., Loup B., Sinclair K.D., Lea R.G., Pocar P., Fischer B., van der Zalm E., Hart K., Schmidt J.S., Amezaga M.R., Fowler P.A. (2010). Effects of environmental pollutants on the reproduction and welfare of ruminants. Animal.

[b0335] Rhind S.M., Kyle C.E., Mackie C., McDonald L., Zhang Z., Duff E.I., Bellingham M., Amezaga M.R., Mandon-Pepin B., Loup B., Cotinot C., Evans N.P., Sharpe R.M., Fowler P.A. (2010). Maternal and fetal tissue accumulation of selected endocrine disrupting compounds (EDCs) following exposure to sewage sludge-treated pastures before or after conception. J. Environ. Monit..

[b0340] Rhind S.M., Kyle C.E., Kerr C., Osprey M., Zhang Z.L. (2011). Effect of duration of exposure to sewage sludge-treated pastures on liver tissue accumulation of persistent endocrine disrupting compounds (EDCs) in sheep. Sci. Total Environ..

[b0345] Sawyer H.R., Smith P., Heath D.A., Juengel J.L., Wakefield S.J., McNatty K.P. (2002). Formation of ovarian follicles during fetal development in sheep. Biol. Reprod..

[b0350] Schmidt D., Ovitt C.E., Anlag K., Fehsenfeld S., Gredsted L., Treier A.C., Treier M. (2004). The murine winged-helix transcription factor Foxl2 is required for granulosa cell differentiation and ovary maintenance. Development (Cambridge, England).

[b0355] Sheffield J.W., O’Shaughnessy P.J. (1989). Effect of injection of gonadotrophin-releasing hormone on testicular steroidogenesis in the hypogonadal (hpg) mouse. J. Reprod. Fertil..

[b0360] Silva E., O’Gorman M., Becker S., Auer G., Eklund A., Grunewald J., Wheelock A.M. (2010). In the eye of the beholder: does the master see the SameSpots as the novice?. J. Proteome Res..

[b0365] Singh M.P., Reddy M.M., Mathur N., Saxena D.K., Chowdhuri D.K. (2009). Induction of hsp70, hsp60, hsp83 and hsp26 and oxidative stress markers in benzene, toluene and xylene exposed Drosophila melanogaster: role of ROS generation. Toxicol. Appl. Pharmacol..

[b0370] Stoop H., Honecker F., Cools M., de Krijger R., Bokemeyer C., Looijenga L.H. (2005). Differentiation and development of human female germ cells during prenatal gonadogenesis: an immunohistochemical study. Hum. Reprod..

[b0375] Uhlenhaut N.H., Treier M. (2011). Forkhead transcription factors in ovarian function. Reproduction.

[b0380] Vantyghem M.C., Vincent-Desplanques D., Defrance-Faivre F., Capeau J., Fermon C., Valat A.S., Lascols O., Hecart A.C., Pigny P., Delemer B., Vigouroux C., Wemeau J.L. (2008). Fertility and obstetrical complications in women with LMNA-related familial partial lipodystrophy. J. Clin. Endocrinol. Metab..

[b0385] Weber A., Pennise C.R., Pring M. (1994). DNase I increases the rate constant of depolymerization at the pointed (−) end of actin filaments. Biochemistry.

[b0390] Wolfe W.H., Michalek J.E., Miner J.C., Rahe A.J., Moore C.A., Needham L.L., Patterson D.G. (1995). Paternal serum dioxin and reproductive outcomes among veterans of Operation Ranch Hand. Epidemiology (Cambridge, Mass.).

[b0395] Yu Z., Fotouhi-Ardakani N., Wu L., Maoui M., Wang S., Banville D., Shen S.H. (2002). PTEN associates with the vault particles in HeLa cells. J. Biol. Chem..

