# Supplementary material for: Exposure to chemical cocktails before or after conception – The effect of timing on ovarian development
Source: Mol Cell Endocrinol. 2013 Aug 25;376(1-2):156–72. doi: 10.1016/j.mce.2013.06.016 (PMC3731555; doi:10.1016/j.mce.2013.06.016)
Supplement: Supplementary data 1 [file mmc1.pdf]

SUPPORTING INFORMATION

Appendix A. Animal Management

At the time of mating, TC animals were transferred for a period of 10 days to a non-experimental, uncontaminated pasture area, commencing 4 days before the introduction of the rams. After this time they were returned to the control pasture; this protocol was designed to minimise contamination of experimental control pastures with pollutant-containing faeces and urine. Similarly, animals previously maintained on control pastures were transferred to the sludge treated pastures 4 days before the introduction of the rams so that the developing embryos were likely to be exposed to diet-associated pollutants from the time of conception. Since all ewes on a given treatment were maintained on the same areas of treated and control pasture, the transfer between pastures of animals of selected treatments involved the gathering of all experimental animals and so they were all subjected to the same, transient stress; the degree of stress involved was considered minimal since the animals were accustomed to regular handling and the rates of conception were normal in all 4 groups.

Appendix B. **Primers for qPCR.**

Gene	Primers
HPRT1	5'-TGGACTAATTATGGACAGGACCG-3'<br>5'-TATAGCCCCCCTTGAGCACA-3'
DDX4	5’-CGAGGGCTGGATATTGAAAA-3’<br>5’-TGCCAGTATTTCCACAACGA-3’
KIT	5'-TGGAGGACTTGCTGAGCTTT-3'<br>5'-GAGGATATTTCTGGCTGCCA-3'
POU5F1	5’-CACCCTGCAGCAAATTAGCC-3’<br>5’-TTGATCGTTTGCCCTTCTGG-3’
MKI67	5’-CAGCATGGGATCCTCCAGAC-3’<br>5’-CCGAGTTTCACCACATCTGC-3’
NOBOX	5’-CTCCTCAACACACTGGCCCA-3’<br>5’-GTCCGAGCGGTACAGGGTG-3’
PTEN	5’-TTTGTGGTCTGCCAGCTAAAGG-3’<br>5’-GGCAATGGCTGAGGGAACTC-3’
BMP15	5’-AAAGCCTTCCCTGTTGCCC-3’<br>5’-GGAGTCGTAGAACCCTGCGC-3’
GDF9	5’-GGGACAACTGGATTGTGGCC-3’<br>5’-GGTGTGAACCGGAGAGCCAT-3’
FOXL2	5’-CCGGCATCTACCAGTACATTATAGC-3’<br>5’-GCACTCGTTGAGGCTGAGGT-3’
FST	5’-ACTCCATTTCGGAAGACACC-3’<br>5’-ATTGGTGGAGGGTTTACCAC-3’
FSHR	5’-ATCACGCTGGAAAGATGGCA-3’<br>5’-AAGATCCAGCCCACCAGCAT-3’
AMH	5’-ACATACCAGGCCAACAACRG-3’<br>5’-TGCATCTTTAGCAGCAGCAC-3’
CYP19A1	5’- AGGTCGGTGCGTTGAGAAGAT-3’<br>5’- AGCGCTCGAGGCACTTGTC-3’
CYP11A1	5’-GTGGCACTTGTACGAGATGGC-3’<br>5’-TCTTGCTTATGTCGCCCTCTG-3’
ESR1	5’-GCCACCAACCAGTGCACAA-3’<br>5’-TTTCCGTATCCCCCCTTTCA-3’
ESR2	5’-AGTGCAAAAATGTGGTGCCC-3’<br>5’-GTTGCGCTCAGACCTTGTGAC-3’
INHBA	5’-ACATCGGGCTCATCCCTCTC-3’<br>5’-GTGGGCACACAGCACGACTT-3’
STAR	5’-AGGTGCTGAGTAAAGTGATCCCTG-3’<br>5’-GCGCTCCACAAGCTCTTCAT-3’
BAX	5’-TTGGACTTCCTTCGAGAGCG-3’<br>5’-GTCACTGTCTGCCATGTGGGT-3’
GSN	5’-CTAATCGTGACCGGCGGAC-3’<br>5’-CAGACCAATAGTTGTCATCCCAGC-3’
CDC45EP5	5’-GACGTCATCGGCCTGTAG-3’<br>5’-CAGACCTTGTCGGTTTATGTAG-3’
CDKN1B	5’-CCTCAAAACAAAAGAGCCAAC-3’<br>5’-TTTACGTCTGACGCCTTCTG-3’

Appendix C. Effects of chemical cocktails in sewage sludge on morphological and endocrine characteristics maternal ewes on day 110 of pregnancy. Values are mean±SEM. Common superscripts denote differences at P<0.05. Paired organs are combined into a single weight.

Treatment groups	Constant exposure profile		Cross-over exposure profile	
	**CC (12)**	**TT (12)**	**CT (11)**	**TC (10)**
**Morphology**				
Live weight (kg)	82±3 [a]	88±2	82±3 [b]	90±1 [ab]
Body condition score	2.3±0.1 [ac]	2.6±0.1 [ab]	2.4±0.1 [b]	2.5±0.1 [c]
Number of fetuses	2.1±0.2	2.2±0.2	2.0±0.2	2.5±0.2
% of male fetuses	40±10	56±9	39±13	51±11
Ovaries (g)	4.8±0.3 [a]	4.8±0.2	4.8±0.3	5.6±0.3 [a]
**Endocrinology**				
Estradiol (pg/ml)	2.5±0.7	0.7±0.1	3.2±1.1	2.8±1.0
Inhibin A (pg/ml)	46±24	46±34	62±40	36±14
FSH (ng/ml)	0.52±0.07	0.37±0.04	0.40±0.05	0.40±0.02
LH (ng/ml)	1.15±0.15 [a]	1.18±0.10 [b]	0.95±0.07	0.83±0.07 [ab]
Testosterone (ng/ml)	0.09±0.01	0.11±0.01 [a]	0.09±0.01 [a]	0.09±0.01
Progesterone (nmol/l)	96±8 [ab]	156±15 [ac]	118±13 [c]	129±8 [b]
