# Supplementary material for: Exposure to chemical cocktails before or after conception – The effect of timing on ovarian development
Source: Mol Cell Endocrinol. 2013 Aug 25;376(1-2):156–72. doi: 10.1016/j.mce.2013.06.016 (PMC3731555; doi:10.1016/j.mce.2013.06.016)

Fig. S1. **Network 1.** Renal and Urological System Development and Function, Reproductive System Development and Function, Embryonic Development .

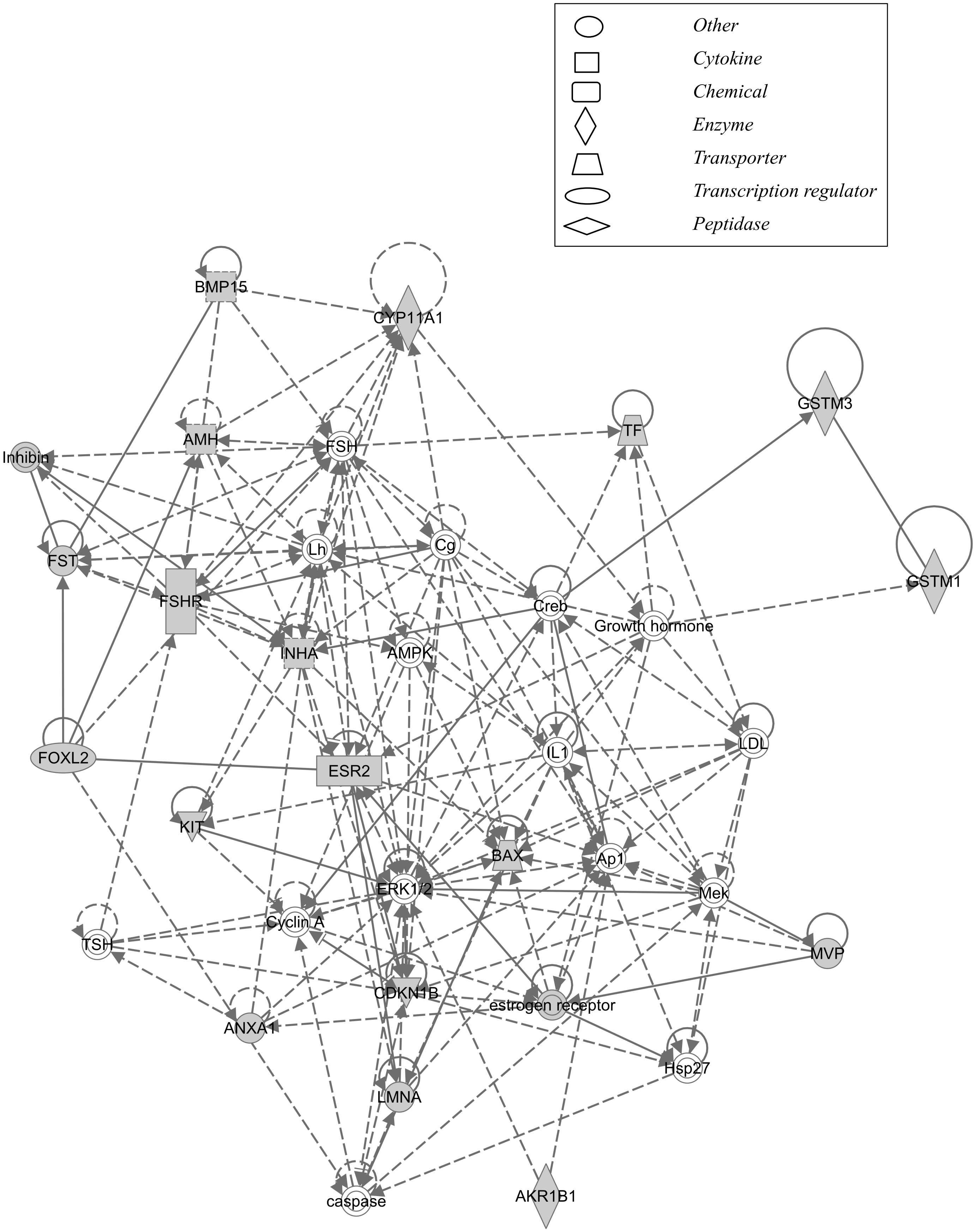

Fig. S2. **Network 2.** Post-Translational Modification, Protein Folding, Organismal Injury and Abnormalities .

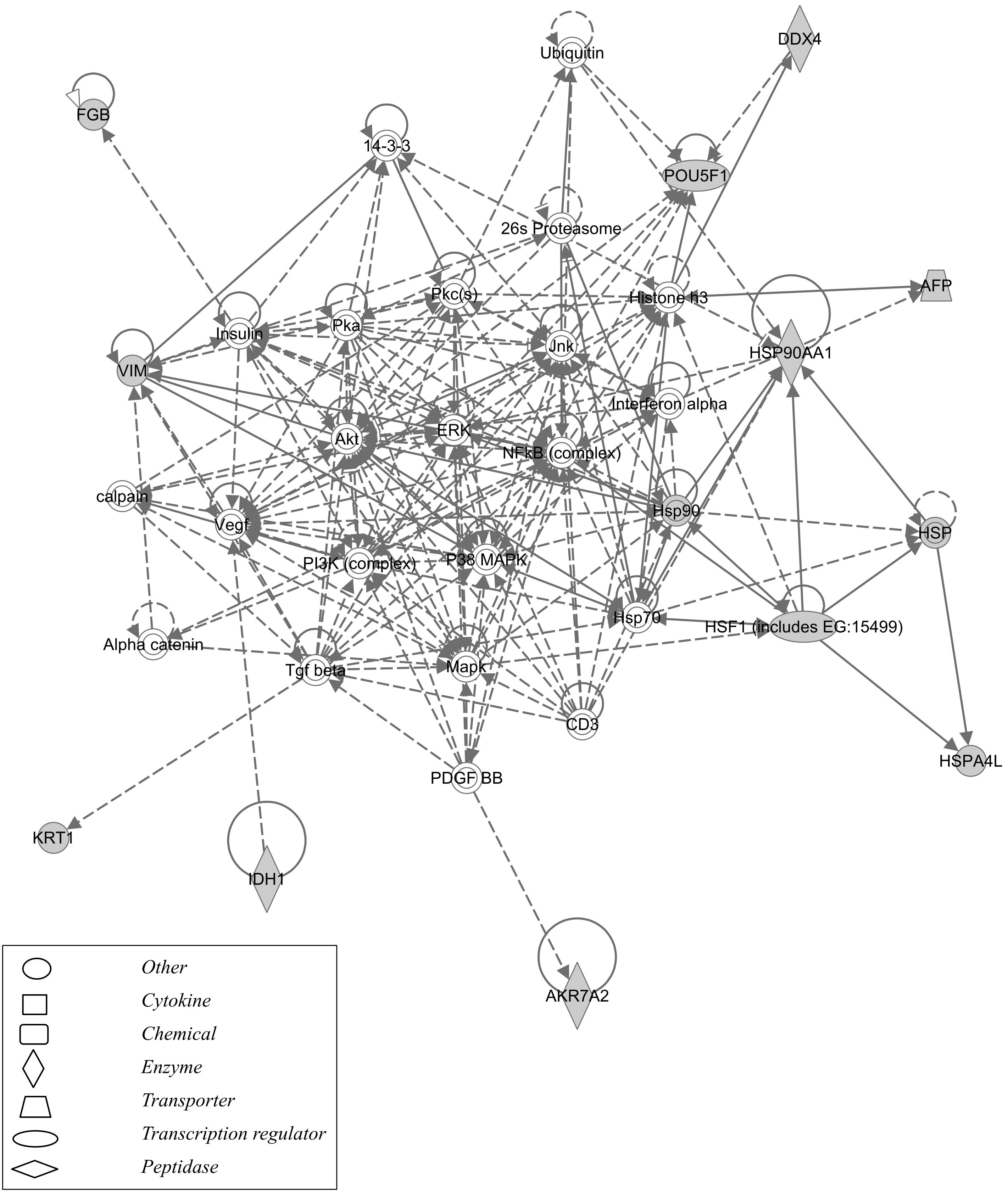

Fig. S3. **Network 3.** Cell Morphology, Cellular Compromise, DNA Replication, Recombination, and Repair .

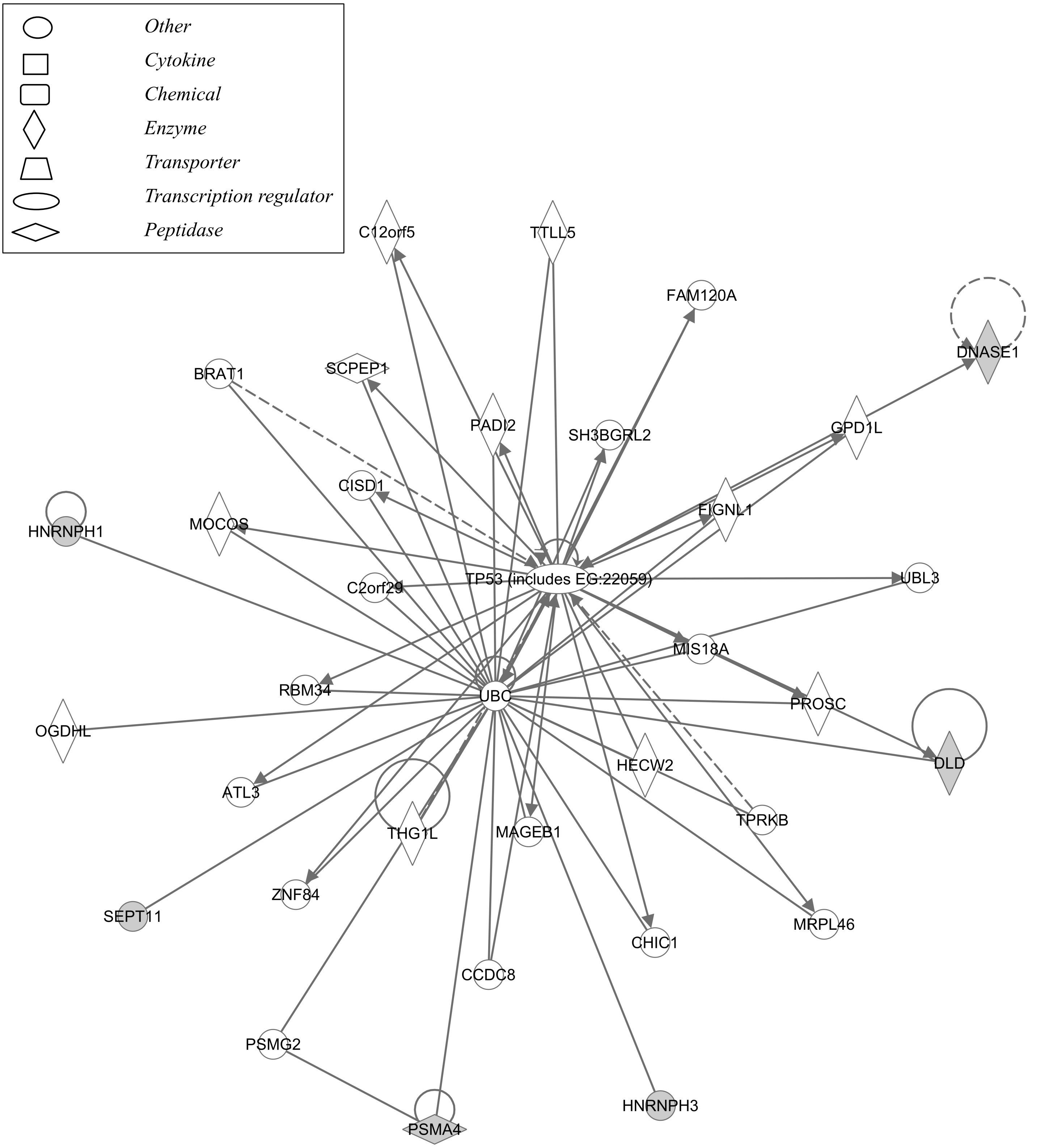

Supplement: Supplementary data 2 [file mmc2.pdf]
